# The Role of Wearable Sensors to Monitor Physical Activity and Sleep Patterns in Older Adult Inpatients: A Structured Review

**DOI:** 10.3390/s23104881

**Published:** 2023-05-18

**Authors:** Gemma L. Bate, Cameron Kirk, Rana Z. U. Rehman, Yu Guan, Alison J. Yarnall, Silvia Del Din, Rachael A. Lawson

**Affiliations:** 1 Translational and Clinical Research Institute, Faculty of Medical Sciences, Newcastle University, Newcastle upon Tyne NE4 5PL, UK; g.l.bate2@newcastle.ac.uk (G.L.B.); cameron.kirk@newcastle.ac.uk (C.K.); rana.zia-ur-rehman@newcastle.ac.uk (R.Z.U.R.); alison.yarnall@newcastle.ac.uk (A.J.Y.); silvia.del-din@newcastle.ac.uk (S.D.D.); 2 Department of Computer Science, University of Warwick, Coventry CV4 7EZ, UK; yu.guan@warwick.ac.uk; 3 The Newcastle upon Tyne Hospitals NHS Foundation Trust, Newcastle upon Tyne NE7 7DN, UK; 4 National Institute for Health and Care Research (NIHR), Newcastle Biomedical Research Centre (BRC), Newcastle University, Newcastle upon Tyne NE1 7RU, UK

**Keywords:** wearables, continuous monitoring, older adults, inpatient, hospital setting

## Abstract

Low levels of physical activity (PA) and sleep disruption are commonly seen in older adult inpatients and are associated with poor health outcomes. Wearable sensors allow for objective continuous monitoring; however, there is no consensus as to how wearable sensors should be implemented. This review aimed to provide an overview of the use of wearable sensors in older adult inpatient populations, including models used, body placement and outcome measures. Five databases were searched; 89 articles met inclusion criteria. We found that studies used heterogenous methods, including a variety of sensor models, placement and outcome measures. Most studies reported the use of only one sensor, with either the wrist or thigh being the preferred location in PA studies and the wrist for sleep outcomes. The reported PA measures can be mostly characterised as the frequency and duration of PA (Volume) with fewer measures relating to intensity (rate of magnitude) and pattern of activity (distribution per day/week). Sleep and circadian rhythm measures were reported less frequently with a limited number of studies providing both physical activity and sleep/circadian rhythm outcomes concurrently. This review provides recommendations for future research in older adult inpatient populations. With protocols of best practice, wearable sensors could facilitate the monitoring of inpatient recovery and provide measures to inform participant stratification and establish common objective endpoints across clinical trials.

## 1. Introduction

Low levels of physical activity (PA) are commonly seen in older adult inpatients [1,2,3,4], with many inpatients spending long periods of time resting in bed, regardless of their diagnosis [3]. This can have a negative impact on functional capacity, such as walking, dressing, toileting and independent transfer (e.g., movement without assistance to get in or out of bed or a chair) [5,6,7,8]. Reduced PA is associated with increased risk of hospital readmissions [9], institutionalisation [8] and mortality [10].

Increased daytime sleep and napping often occurs in older adults and is frequently associated with disrupted night-time sleep [11,12]. Older inpatients commonly experience frequent night-time awakenings and disturbance [13,14] due to factors such as noise, lighting, medical care interruptions and pain [14,15,16]. Loss of sleep has been linked to increased risk of poor health outcomes [16] including high blood pressure [17], delirium [18], reduced immunity [19] and mortality [20]. Disrupted rest–activity patterns (circadian rhythm) such as fragmented night-time sleep and greater daytime sleep are also linked with negative health outcomes in older inpatients [21,22]. Measuring both PA and sleep/circadian rhythm (SCR) constructs concurrently allows a more nuanced approach for studying hospital-based recovery patterns.

Standard measures of physical activity and sleep constructs, such as activity logs and sleep diaries, have inherent recall and reporting bias [23,24] particularly evident when used in older adults with cognitive impairment [24]. Polysomnography (PSG) is the criterion measure for sleep analysis; however, this only provides information for short periods in controlled environments. This method is also costly, requires trained staff and relies on patient compliance (e.g., for the overnight attachment of electrodes and wires) and hence is generally impractical for hospitalised patients [25]. Wearable sensors are small, non-invasive and provide a continuous and objective method of measuring both PA and SCR outcomes collectively [26]. A wide range of objective metrics can be obtained, including the frequency, intensity and duration of physical activity, to further constructs such as sedentary behaviour, rest–activity and sleep patterns [27]. Wearable sensors have been used in large community-based epidemiologic studies [28,29] and in various clinical populations such as: stroke patients [30], older adults within residential care facilities [31,32] and in individuals with cognitive impairment [31,32,33]. However, hospitalized older adults are often frailer and more unwell than community-based adults, which questions the feasibility of using wearable sensors in this population.

Numerous studies have used wearable sensors in inpatient populations; however, there is no consensus as to protocol use (e.g., which sensors to use, the number, body position, duration of wear) or optimal measures. This review aimed to consolidate the methods of studies that used wearable sensors to measure unscripted (free living) physical activity and sleep behaviour in the hospital setting with all older adult subject populations. Specifically, this review aimed to identify and categorise the study population and to report on the sensor placements, sensor models, monitoring protocol and outcome measures. Secondly, this review aimed to explore the acceptability of the sensors in terms of compliance, and usability in older inpatients.

## 2. Materials and Methods

### 2.1. Search Method

Five databases were searched from earliest records to February 2021: PubMed, Ovid Embase, Scopus, Web of Science and Cochrane database. The reporting of this systematic review was guided by the standards of the Preferred Reporting Items for Systematic Review and Meta-Analysis (PRISMA) Statement [34]. The search strategy was developed in PubMed using medical subject headings (MeSH) based on the key concepts of the research question “wearable sensor”, “hospital” and “older adult” (Supplementary Materials, Table S1). To ensure a comprehensive search a list of synonyms was identified from the MeSH terms and from previous reviews. Duplicates were removed from the compiled articles and the titles and abstracts were then reviewed independently by two reviewers (GB and CK). If it was unclear if the paper met the inclusion criteria, the full text was reviewed.

### 2.2. Inclusion and Exclusion Criteria

Inclusion criteria included inpatients, defined as a facility away from the participants own home/community setting which provided a level of service/specialised care, as either elective or emergency admission, with an overall study mean or median age of ≥65 years, or a clearly defined subsample of the study being of ≥65 years. The study criteria included objectively assessed, non-protocolled wearable sensor outcomes to measure activity, movement, or sleep. Studies were excluded if they used scripted activities. We did not exclude studies based on healthcare condition, including participants with dementia, or prognosis. Articles that focused on day cases (<24-h admissions) or long-term rehabilitation, defined as expected stay of over two weeks, were excluded. In studies which included community dwelling settings, such as older adult residential care settings or repeated measures to include the home environment, only the hospital setting outcomes were used for data extraction. Articles were excluded if the sample size was n ≤ 10, written in other languages than English, were conference abstracts, case reports, literature reviews, meta-analysis, grey literature or study protocols.

### 2.3. Data Extraction

Data were independently extracted by two reviewers (GB and CK) using a standardised data extraction form. Key outcomes included the study setting, clinical population, type and model of sensor, body placement, participant acceptability/compliance and the sensor measures derived. Any discrepancies in screening or extraction were resolved by an independent reviewer (RAL or RZUR). Due to the mixed study designs included in this review, a customised quality appraisal form was produced adapted from Downs and Black [35] for health interventions and the National Institutes of Health (2014) Quality Assessment Tool for Observational Cohort and Cross-sectional Studies [36]. Assessments were completed independently by GB and CK and an average quality score was derived (Supplementary Materials, Table S1).

## 3. Results

### 3.1. Search Yield

**Figure 1:**
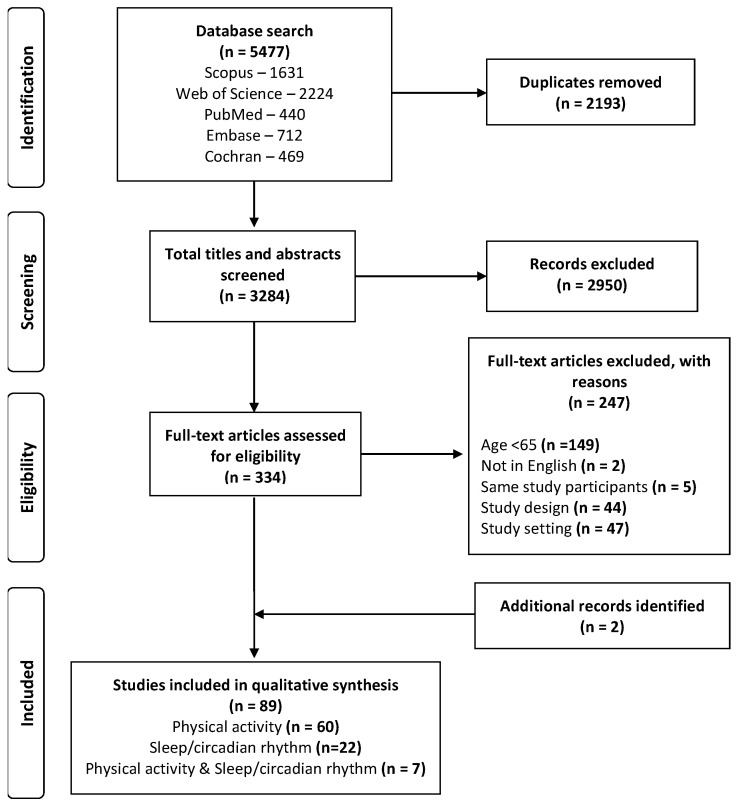
PRISMA flow diagram.

The search yielded 5477 papers (Figure 1); after duplicates were removed 3284 papers were screened by title and abstract, 2950 papers met exclusion criteria and the full text was reviewed in the remaining 334 articles. From the full text review, n = 247 articles were excluded due to study participants were less than 65 years old (n = 149), the full text not being available in English (n = 2), the articles comprised the same study participants (n = 5), protocolled/scripted sensor activity measures were included (n = 44) and study setting involved long term rehabilitation or an expected stay of less than 24 h (n = 47). In addition, two further papers were identified from reviewing the reference lists of the studies [37,38]. The final sample comprised 89 articles, 60 articles on PA measures only, 22 articles on SCR measures only and seven articles which included both PA and SCR measures. To provide a framework for data synthesis, the seven studies that report on both PA and SCR outcomes were considered separately. A summary of all included studies with PA outcomes is presented in Appendix A, Table A1 and all studies with SRC outcomes in Table A2.

### 3.2. Study Characteristics

Of the 89 articles, the quality review identified 6 studies that were rated excellent, n = 60 studies rated as good, n = 22 rated as moderate and one study rated as poor [39], all articles were included in this review (Supplementary materials, Table S2). The review captured 74 (83%) observational studies (Appendix A; Table A1 and Table A2), 11 (12%) studies used an experimental design [40,41,42,43,44,45,46,47,48,49,50] and 5 (6%) studies were randomised controlled trials [40,44,46,48,49]. Four were validation studies in which wearable sensors were correlated against PA questionnaires [51], clinical scales [52], treadmill [53] or polysomnography [54].

Studies were conducted across 20 countries (Appendix A; Table A1 and Table A2), the majority were in the United States of America (n = 19) followed by Denmark (n = 10) and Japan (n = 9). Studies included 40 emergency admissions [4,9,10,22,33,46,55,56,57,58,59,60,61,62,63,64,65,66,67,68,69,70,71,72,73,74,75,76,77,78,79,80,81,82,83,84,85,86,87,88], 18 elective [37,38,39,41,44,47,89,90,91,92,93,94,95,96,97,98,99,100], 6 mixed [52,53,101,102,103,104], 5 rehabilitation [51,105,106,107,108] and in n = 20 it was unclear [3,40,42,43,45,48,49,50,54,109,110,111,112,113,114,115,116,117,118,119].

Participants came from 11 study populations (Appendix A; Table A1 and Table A2): older adults (n = 17: PA n = 16, SCR n = 2) [3,4,10,45,46,59,60,61,62,63,101,106,107,113,114,115,116], mixed admissions (delirium and dementia) (n = 12: PA n = 8, SCR n = 7) [33,38,42,43,65,66,67,68,93,94,95,119], orthopaedic surgery/fractures (n = 12: PA n = 11, SCR n = 2) [41,52,69,70,71,72,73,96,97,98,99,102], cardiac medical/surgical patients (n = 10: PA n = 7, SCR n = 4) [39,50,53,74,75,100,103,104,117,118], mixed medical admissions (n = 9: PA n = 3, SCR n = 6) [9,22,55,56,57,58,105,111,112], stroke patients (n = 9: PA n = 8, SCR n = 1) [79,80,81,82,83,84,85,86,87], respiratory patients (n = 6; PA n = 6) [40,51,76,77,78,88], oncology (n = 6: PA n = 4, SCR n = 3) [37,47,90,91,109,110], patients in intensive care (n = 4: PA n = 1, SCR n = 3) [48,49,54,64], other surgery (n = 3: PA n = 2, SCR n = 1) [44,92,108] and patients with Parkinson’s disease (n = 1: PA n = 1) [89].

### 3.3. Sensor Placement

The wrist was the most common sensor placement across all studies (n = 40: PA n = 21, SCR n = 27) (Table 1), followed by the thigh (PA n = 21) [3,4,40,41,47,59,67,69,71,73,75,80,81,82,86,87,101,102,106,107,114] and ankle (n = 16: PA n = 16, SCR n = 2) [3,9,10,39,45,49,53,61,63,68,69,70,75,79,112,113]. A single sensor was used in 52 (78%) of the PA studies (Appendix A; Table A1). Two sensors were used in (n = 12) PA studies, this included both wrists [83,84], wrist and ankle [61], thigh and ankle [3,69], waist and ankle [53], wrist and thigh [67], chest and thigh [40,47,81] and lower leg and thigh [4,114]. In two studies, three sensors were placed this included the wrist, ankle and arm [68] and the thigh, ankle and waist [75]. One study placed five sensors on each participant to include bilateral wrists and ankles and hip [79].

The wrist was the sole and preferred sensor attachment site in all but four of the SCR studies (Table 1). Chen et al. [49] used the wrist or ankle for securement, Macfarlane et al. [56] used the upper arm, Enomoto et al. [57] secured the sensor at the waist and Davoudi et al. [68] used three sensors on the wrist, ankle and arm.

### 3.4. Sensor Model

Twenty-five different sensor models were identified (Supplementary material, Table S3). All but four studies [40,43,50,74] described the make or model of the sensor used. All studies used sensors that comprised of an accelerometer (to measure linear acceleration) apart from Stubbs et al. [43] reporting the use of a pedometer (providing no further detail). The most commonly used sensor model for the PA studies was ActivPAL identified in 15 studies [47,59,67,71,73,75,80,81,82,86,87,101,102,106,107] followed by Actigraph (n = 6) [44,46,58,67,68,94] and Fitbit (n = 5) [39,53,62,91,96]. For the SCR identified studies the most commonly used includes: Motionlogger—Ambulatory Monitoring Inc., Ardsley, NY, USA, (n = 9) [55,65,85,95,105,109,110,115,118], Actiwatch (Philips Respironics) (n = 9) [37,48,54,97,98,104,111,116,119] and Actigraph (n = 3) [49,68,92] Actiwatch (Cambridge Neurotechnology, Cambridge, UK) (n = 3) [22,38,93], SenseWear Armband (n = 1) [56] and Lifecorder (n = 1) [57]. Two studies reported using a sensor with an added gyroscope, Thorup et al. [53] using a Shimmer 3 and Denkinger et al. [52] using a Physilog activity monitor (Appendix A, Table A1). Six studies used sensor models that provided accessible raw accelerometer data (gravitational acceleration rather than brand defined units), this includes: Axivity [69,70,114], Misfit Shine [69], GeneActive [60], Shimmer [53] and Physlog [52].

### 3.5. Monitoring Protocol

In PA studies, sensors were protocolled to be worn continuously in 56 (84%) studies (Appendix A; Table A1). Data were collected or analysed for daytime hours only in ten (15%) studies [43,51,52,73,78,87,88,89,90,117] with the continuity of monitoring not defined in one study [39]. The overall sensor wear-time period varied between the PA studies, with wear time defined as simply during admission in fifteen (22%) studies [9,41,62,75,89,90,91,94,96,101,108,112,116,117,118]. More specifically, only 1 daytime period (≤12 h) was defined in 3 studies [52,73,88], 24 h (or at least) of wear time was defined in 13 (19%) of the PA studies [10,40,53,58,60,61,63,67,69,83,86,106,107], 5 studies specified 2 days [78,82,84,91,114], 4 days were defined in 1 study [103], 3 days (or at least 3 days) were defined in 10 studies [43,44,51,66,70,76,80,81,98,109], 5 days in 3 studies [78,100,102] and 1 study each for 8 days [99], 10 days [33] and 14 days [42]. The monitoring period was not clearly defined in two PA studies [39,87]. A maximum wear time was reported in 16 (24%) studies (range 3–20 days) [3,4,38,45,46,47,53,61,64,68,71,72,77,79,80,113].

For SCR studies, the sensors were protocolled to be worn continuously in 24 (83%) studies (Appendix A; Table A2). Night-time only data collection occurred in five (17%) of the SCR studies [37,49,54,56,65]. Eight studies defined wear time/analysis period as during admission [22,48,50,65,111,116,118,119] with five studies providing a wear time cut off point (range 6–10 nights) [33,38,68,92,93]. More specifically, 24 h/one night was defined in six SCR studies [37,54,55,56,74,98], two nights defined in three studies [49,57,97], three nights in four studies [85,95,109,110], five nights in one study [104] and seven nights in two studies [105,115].

### 3.6. Physical Activity Outcomes

Twenty-seven separate physical activity definitions were identified across the PA studies (Table 2) (Supplementary material, Figure S1). The volume of PA, defined as the time spent in PA for a specified time frame, was included in 65 (97%) of the identified studies. Number of steps/step counts was the most common outcome measure across studies (n = 32, 48%) [9,10,39,43,44,45,46,47,51,53,61,62,63,66,72,73,74,75,76,77,78,82,87,90,91,99,100,102,103,108,112,113], followed by time spent standing and/or walking (n = 25, 37%) [3,4,40,41,47,52,59,61,63,66,67,71,73,75,78,80,81,82,86,87,88,102,106,107,114] and time spent lying and/or sitting (n = 16, 24%) [3,4,40,47,66,73,75,78,80,81,82,86,87,88,106,114]. The intensity (metabolic demand of PA) was included in 15 (22%) of the studies. Active minutes defined by various metrics was the most common intensity outcome identified in seven studies (10%) [46,58,60,61,69,70,102], followed by minutes of moderate or above intensity (n = 4, 6%) [76,91,100,102], MET minutes active per day/week (n = 3, 4%) [76,100,108] and active energy expenditure/calories per day (n = 3, 4%) [51,89,103]. The distribution of PA per day (pattern) was included in 18 (27%) studies, with nine (13%) [38,42,59,64,68,77,94,118] identified as night-time activity outcomes, seven (10%) [38,42,59,64,68,94,118] as day-time activity outcomes and five (7%) [39,41,79,81,89] as daily variation outcomes.

### 3.7. Sleep and Circadian Rhythm Outcomes

Twenty-five separate sleep and circadian rhythm definitions were identified across the SRC studies (Table 3) (Supplementary material, Figure S1). Total sleep time at night was the most common sleep outcome, identified in 22 (76%) of the studies [37,48,49,54,55,56,57,65,68,74,85,92,93,95,97,98,104,105,109,111,116,119], followed by sleep efficiency (n = 14, 48%) [37,48,54,55,56,57,74,92,97,104,109,111,116,119]. Wake after sleep onset (WASO) [37,48,74,85,93,95,109,119], total sleep time during the day [68,74,85,98,104,105,109,115] and number of awakenings [33,37,49,54,55,85,95,105] were each identified in eight (31%) of the studies.

The most common circadian rhythm outcome was activity amplitude identified in three (10%) studies [22,38,68] (Table 3). Each of the remaining circadian rhythm outcomes were identified in one study: Cosinor parameters (acrophase, amplitude, mesor, percent rhythm) based on fitting each 24 h data with a (co)sine curve [118], Highest mean activity in any ten hours (M10), Lowest mean activity in any five hours (L5) [68], restlessness index (RI) [38], interdaily stability (IS), intradaily variability (IV) [22], rest activity counts less than the median activity during the rest span (I < 0) [49] and 24 h autocorrelation coefficient R_24_ [109].

The criteria defining the sleep period and time for the intention to sleep varied between studies, this included a sleep diary/log [37,92,93,95,97,109,110,111,116] or, time of light on/off to define bedtime and final awakening [56,98,119]. Sleep periods were set by the experimenter in five studies [55,65,74,85,104] or reported by observation in three studies [49,54,57]. In three studies, definitions were unclear or ill-defined [48,50,105]; or outcomes were directly provided by proprietary software [33,49].

### 3.8. Acceptability and Tolerance

Participant views and feedback regarding wearing the sensors were not reported in any of the SCR studies and in only three of the PA studies [61,75,114] (Appendix A; Table A1 and Table A2). The comfort of the sensors and the compatibility with hospital technology/medical care were the themes reported from a semi structured interview [75]. Using feedback from a questionnaire, Lim et al. [61] reported that the acceptability for both the wrist and ankle sensor was high, with 96% of participants tolerating the wrist sensor and 83% tolerating the ankle sensor. One study used a short participant questionnaire and found no discomfort or sleep disruption when using a thigh and lower leg sensor, with 96% of the participants agreeing to wear the sensors again [114].

**Figure 2:**
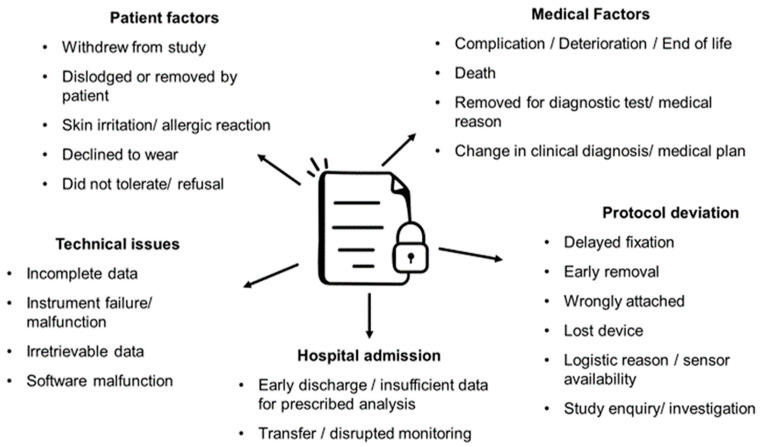
Reasons for missing, incomplete, or withdrawn sensor data across studies.

Recruitment information was provided in 34 (38%) of the identified studies (recruitment rate: range 25–100%), with 61 (69%) of the studies providing information for retention (retention rate: range 47–100%) (Appendix A; Table A1 and Table A2). Sources of missing or incomplete sensor data were reported with varying levels of detail and described in some capacity for 69 (78%) of the studies (Figure 2). Most commonly reported reasons for missing data were participant withdrawing from the study reported in 17 (19%) [3,40,42,43,45,50,52,53,59,66,80,86,87,88,91,93,100] studies, sensor malfunction/instrument failure reported in 16 (18%) [41,44,46,73,77,79,80,84,86,90,94,95,96,111,112,116] studies, early discharge in 15 (19%) [9,44,45,47,48,50,52,53,78,81,87,88,89,93,114] studies and also death in 15 (19%) [22,37,39,45,46,62,64,71,78,85,86,87,91,93,104] studies. Skin irritation was reported in six (7%) studies [40,50,76,82,91,102], with sensors reported as lost in four (5%) studies [39,67,106,112].

## 4. Discussion

To our knowledge, this is the first study to provide a comprehensive overview of the use of wearable sensors in older adult inpatients. Studies included a range of older adult hospital populations using heterogenous methods, including a variety of sensor models, placement and outcome measures. Wearable sensors have been used mainly to derive PA outcomes in older adult inpatients as opposed to the more limited number of studies addressing sleep or circadian rhythm outcomes. The inclusion of both PA and SCR outcomes in this review has highlighted the paucity of studies that derive both outcomes concurrently.

### 4.1. Sensor Placements

A variety of sensor body locations were identified, with the wrist or thigh location each being used in a third of PA studies and the wrist location predominantly used in all but three of the SCR studies. The wrist location was the preferred sensor placement for sleep measures in this review, and has historically been used as it is more reflective of movements of the total trunk and less of movements involved in performing specific tasks [120]. Sensors provide measures of movement reflective of the area they are secured, which is important to consider when research planning. For example, to characterise movement of the impaired and non-impaired arm of stroke patients, Gebruers et al. [84] and Iacovelli et al. [83] secured sensors to both wrists to capture arm movement. When research design is focused on general mobility, e.g., magnitude or intensity of physical activity, sensors are commonly placed on the trunk of the body close to the centre of mass to represent full body movement (rather than general limb movement) [121]. In this review the wrist position was commonly used to provide general mobility measures, relying on arm movement to estimate activity and sedentary behaviour. This may pose significant challenges for measurement accuracy, particularly for quantifying sedentary behaviour [122]. However, in older inpatients who are generally frail and unwell, wrist placement may be less intrusive and a more practical option.

In over a half of studies that reported walking outcomes, the sensor was placed at the thigh or ankle, which has historically been favoured as it is close to the impact site when walking [123]. Four studies each used the wrist for step count estimation [46,62,91,96] or the upper arm [51,76,77,100] which may not provide accurate measures in people with limitations in mobility or slow walking speed [124], as commonly seen in older inpatients. Further locations were identified in the review to quantify steps which included the lower back [66,78], waist [44,90,99,103,108,117] and hip [72] each using different sensor models and protocols.

The sensor was placed on the thigh to provide postural outcomes such as time spent sitting and standing, and sit to stand transitions. Only two studies that used a sensor placed at the thigh focused on step count and activity intensity with no reported postural outcomes [69,102]. A combination of sensor placements; thigh and trunk [40,47,81], thigh and ankle/lower leg [3,4] and thigh, trunk and ankle [75] were used to provide orientation information to help distinguish between time spent sitting and lying and to characterise postural transitions. Securing more than one sensor enabled Evenson et al. [67] to capture wrist movement while sitting to represent restlessness in older adults with delirium. Sensor placement combinations has the potential to provide richer data allowing for further activity characterisation; however, further research is needed into the acceptability and compliance of using wearable sensors in different body locations.

### 4.2. Sensor Models

Accelerometer-based sensors were predominantly used for physical behaviour monitoring across studies with twenty-five sensor models identified. This is greater than the seven sensor models identified in a previous review [125]; however, this was limited to acute general medical inpatients, excluding specific neurological conditions (such as stroke and Parkinson’s disease). The Activpal was used in almost a quarter of studies deriving PA outcomes, while the Motionlogger and Actiwatch were each used in a third of the sleep/circadian rhythm studies. Two studies in this review included sensors with an additional gyroscope [52,53], these are known as inertial measurement units (IMU’s) and provide a measure of angular velocity [126]. With advances in technology, newer generations of sensors exist which accounts for the many sensor models and versions identified in the review. Many sensor manufacturers provide proprietary software with specific algorithms to derive physical behaviour measures; however, most are not made publicly available. This provides a challenge comparing measures across studies. This review identified six studies that used sensors able to provide raw acceleration data [52,53,60,69,70,114]. Using sensors that allow access to raw data compatible with open-source software is advised as it could potentially standardise analysis across studies to allow more meaningful comparisons. Wrist derived raw accelerometer data from the sensor models GENEActiv, ActiGraph and Axivity can be processed using GGIR, for example, which is an open source R package that can generate both physical activity and sleep outcomes concurrently [121].

### 4.3. Sensor Outcomes

A number of wearable sensor outcomes were identified with time spent stepping/step count identified in almost half of the physical activity studies. Such ambulatory measures may not be the most insightful or appropriate, as many older adult inpatients commonly spend limited time mobile [3]. If able to mobilise, older adult inpatients generally do so with a slow walking speed in which steps may not be accurately identified by many wearable sensors [127]. Furthermore, the use of walking aids and devices could hinder step count recognition [128]. Characterising various body positions allows for behaviour that is seen more commonly at the patient bedside, such as transferring from lying in bed to sitting in a chair or sitting to standing and may provide a more authentic activity measure for many older inpatients. Over one third of the PA studies in this review reported time spent upright (either alongside step measures or as a stand-alone measurement) and as expected in older adult inpatients physical activity levels are low. Postural recognition can also provide a measure for non-ambulatory or sedentary behaviour. Time spent lying or sitting was reported in 24% of the PA studies. This is an area of increasing interest due to the link established between sedentary behaviour (rather than activity) and poor health outcomes [129,130].

Research based on community dwelling older adults suggests that the pattern of activity and sedentary time and how this is accumulated (i.e., prolonged vs. shorter bouts) is also of importance for health outcomes [131]. This review found only 27% of the PA studies included measures associated with the distribution of physical activity, defined as total activity or time in postural positions, over specified time periods such as night, day, or hourly. Only one study by Norvang et al. [81] provided outcomes relating to bouts of activity and reported the duration and time for sitting, lying and upright bouts. Further pattern-based measures of physical behaviour in older adult inpatients may provide richer information to add to the growing literature.

Sleep disruption and periodical awakenings is common in many older adult inpatients and are associated with various negative health outcomes including high blood pressure [17], delirium [18], reduced immunity [19] and mortality [20]. Only 29 studies reported measures based upon sleep or rest activity patterns (circadian rhythm outcomes). Twenty-five separate measures were reported, with total sleep time at night and sleep efficiency (percentage of time asleep between being in bed and final awakening) being the most used. The criteria defining these measures differed greatly between studies, with the start of the sleep interval and the intention to sleep conceptualised and measured in various ways, ranging from diary recorded time to bed [37,92,93,95,97,109,110,111,116] or lights off [56,98,119], to times set by the experimenter [55,65,74,85,104], observations by clinical staff [49,54,57] and times derived by software algorithms [33,49]. It has been suggested that such loosely defined criteria and measures can adversely affect behavioural clinical interventions and sleep outcome research [132]. A standard reporting method which establishes intention to sleep is needed to allow for more meaningful comparisons across studies.

A focus on rest activity patterns (circadian rhythm measure), provides a broader measure of physical behaviour and relates to the degree of synchronisation of rest and activity to the 24-h cycle. Greater daytime rest episodes alongside pronounced activity at night suggests a disrupted rest–activity pattern (circadian rhythm) and has been linked to negative health outcomes [21,22]. Only six studies reported circadian rhythm measures in this review, with activity amplitude (the difference between the least active 5-hr period and the most active 10-hr period within each 24-hr period) most common and reported in three of the studies. Implementing such measures moves away from the sleep classification concerns associated with low levels of movement seen in older inpatients; in which motionless wake could be misclassified as sleep [133,134], providing more valid outcome measures.

Sensors were protocolled to be worn continuously in 84% of the studies in this review; however, only 8% reported both PA and SCR outcomes. With the building evidence to suggest an interdependent relationship between PA and SCR [135,136] deriving both measures simultaneously in future studies may provide a more nuanced approach to behaviour monitoring and warrants further investigation.

### 4.4. Acceptability

Limited information was reported regarding the acceptance and compliance of wearing the sensors. Recruitment rates were reported in only 38% of studies, with 69% reporting retention rates. However, caution is advised if using retention rates to inform study planning, as this was defined differently across studies, depending on whether any missing sensor data were considered to compare between the number of participants recruited to analysed. Direct participant feedback was reported in only three studies [61,75,114]. This feedback suggests that wearables were well tolerated in older inpatients; however, this did not include feedback from individuals with cognitive impairment which can be a common exclusion criterion. Reasons for missing, incomplete or withdrawn sensor data were varied, but most frequently was participant withdrawal from the study, alongside factors relating to the hospital setting, such as early discharge and death. Of particular interest, 18% of studies reported sensor malfunction/instrument failure as reasons for missing data. These factors are particularly relevant for research planning.

### 4.5. Limitations

To our knowledge, this review is the first study to provide a comprehensive overview of the use of wearable sensors to measure both physical activity and sleep behaviour in older adult inpatients, yet this should be considered in light of several limitations. Firstly, we excluded articles not available in English. Therefore, relevant papers may have been missed; however, we undertook a comprehensive and rigorous search of the literature (Supplementary Materials, Table S1) and believe this review includes the key findings in this area.

Secondly, the sample size of the studies ranged from eleven to n = 777. A small number of studies investigated sleep/circadian rhythm outcomes compared to PA with only seven studies investigating both PA and sleep/circadian rhythm concurrently. With non-uniformity of study numbers across groups it is difficult to compare and generalize study findings.

We included a quality assessment to ascertain the methodological quality, and rigor of the studies included. However, we acknowledge the large methodological heterogeneity in included studies, making it difficult to compare the validity across studies. Although most studies did show moderate to good quality, the reporting of the data processing methods and addressing the validity of the sensor outcomes varied. Many studies do use proprietary software for data processing and manufacturers generally do not publish their algorithms, this may explain the limited detail reported. However, when a manufacturer allows for pre-programming input (e.g., sampling frequency and epoch length), this should be reported for reproducibility [137]. In addition, different sensor may be limited due to battery life which may impact on the duration in which studies could collect data. Caution is needed when assessing the validity of the sensor outcomes, as validation studies are commonly based on healthy populations and may not adequately represent older adults particularly in inpatient settings [138]. Cut points used to establish physical intensity levels, such as light or moderate energy expenditure and sleep wake thresholds for sleep analysis, are population specific [139] and are dependent on physiological factors, attachment site and processing protocols [138]. As demonstrated in this review this can vary greatly in the older adult hospital population.

## 5. Conclusions

In summary, this review highlights the heterogenous methods used for monitoring older adult inpatients including a variety of sensor models, placement and outcome measures, making it a challenge to compare findings across studies. A limited number of studies measure both physical activity and sleep/circadian rhythm constructs concurrently, and this requires further attention. The use of a wrist sensor has the potential to provide both sleep measures and rest activity patterns that have not been readily investigated in older inpatients. Combining a wrist sensor with a sensor positioned to allow postural differentiation, such as thigh or lower back, would provide richer data for physical behaviour characterisation in older inpatients. The use of sensors that provide access to raw data compatible with open-source software is advised, as this would help to standardise analysis across studies to allow more meaningful comparisons. Further research should include population specific validation studies to help inform standard guidance on which sensors to use and the most appropriate body locations. Although sensor model and placement site are dependent on the outcome measures required, consideration must be given to the study population. Finally, further research is needed to explore the acceptability and compliance of the sensors worn at different body locations, which is crucial for future research planning. With standardised protocols of best practice, wearable sensors could facilitate the monitoring of older adult inpatient recovery and rehabilitation by providing measures to inform participant stratification or to establish common objective endpoints in future clinical trials.

## Figures and Tables

**Table 1:** The sensor placement sites, and physical behaviour outcomes identified in the review.

		Sensor Position										Physical Activity						SCR	
Study Population Clinical Group	Author	Wrist	Thigh	Ankle	Waist	Upper Arm	Chest	Lower Back	Hip	Lower Leg	NR	General Mobility	Walking	Body Positions	Transitions	Arm Movement	Sedentary	Sleep	Circadian Rhythm
Stroke	(Askim et al., 2013) [86]		** 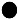 **											** 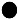 **	** 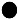 **		** 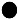 **		
	(Bakken et al., 2012) [85]	** 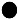 **																** 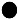 **	
	(Gebruers et al., 2013) [84]	❷														** 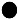 **			
	(Iacovelli et al., 2019) [83]	❷														** 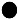 **			
	(Kerr et al., 2016) [82]		** 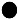 **										** 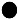 **	** 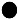 **			** 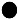 **		
	(Kunkel et al., 2015) [87]		** 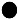 **										** 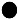 **	** 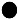 **			** 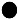 **		
	(Norvang et al., 2018) [81]		** 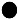 **				** 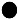 **							** 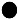 **			** 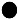 **		
	(Sheedy et al., 2020) [80]		** 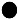 **										** 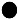 **	** 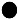 **			** 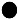 **		
	(Strommen et al., 2014) [79]	❷		❷					** 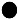 **			** 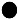 **					** 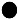 **		
Respiratory	(Borges and Carvalho, 2012) [78]							** 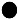 **					** 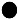 **	** 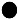 **			** 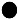 **		
	(Dall et al., 2019) [40]		** 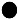 **				** 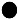 **						** 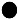 **	** 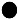 **			** 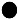 **		
	(Donaire-Gonzalez, 2011) [51]					** 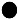 **						** 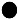 **	** 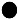 **				** 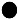 **		
	(Orme et al., 2019) [77]					** 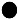 **							** 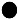 **						
	(Pitta et al., 2006) [88]										** 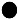 **	** 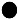 **		** 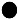 **			** 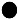 **		
	(Tsai et al., 2016) [76]					** 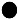 **						** 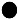 **	** 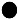 **				** 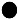 **		
Cardiac	(Amofah et al., 2016) [104]	** 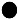 **																** 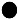 **	
	(Cook et al., 2013) [39]			** 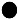 **									** 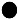 **						
	(Floegel et al., 2019) [75]		** 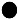 **	** 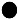 **	** 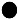 **								** 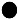 **	** 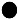 **			** 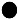 **		
	(Gimenez et al., 2017) [50]	** 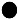 **																** 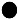 **	
	(Izawa et al., 2015) [103]				** 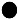 **							** 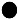 **	** 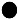 **						
	(Mungovan et al., 2017) [100]					** 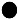 **						** 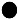 **	** 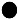 **						
	(Redeker and Wykpisz, 1999) [118]	** 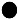 **										** 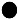 **							** 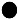 **
	(Takaesu et al., 2015) [74]	** 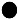 **																** 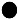 **	
	(Takahashi et al., 2015) [117]				** 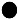 **								** 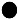 **						
	(Thorup et al., 2017) [53]			** 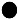 **	** 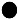 **								** 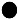 **						
Orthopedic	(Davenport et al., 2015) [73]		** 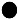 **										** 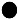 **	** 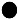 **			** 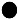 **		
	(Denkinger et al., 2014) [52]						** 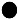 **						** 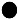 **	** 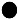 **					
	(Hayashi et al., 2018) [99]				** 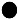 **								** 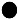 **						
	(Keppler et al., 2020) [72]								** 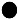 **				** 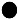 **						
	(Krenk et al., 2013) [98]	** 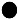 **										** 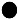 **						** 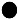 **	
	(Kronborg et al., 2016) [71]		** 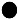 **											** 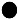 **	** 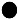 **				
	(Marsault et al., 2020) [70]			** 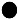 **								** 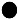 **					** 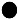 **		
	(Miller et al., 2015) [97]	** 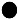 **																** 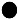 **	
	(Peiris et al., 2013) [102]		** 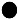 **									** 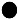 **	** 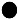 **				** 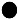 **		
	(Schmal et al., 2018) [69]	** 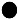 **	** 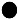 **	** 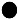 **								** 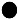 **					** 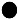 **		
	(Twiggs et al., 2018) [96]	** 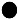 **											** 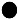 **						
	(van Dijk-Huisman et al., 2020) [41]		** 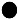 **											** 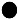 **					
Mixed (Delirium, Dementia)	(Davoudi et al., 2019) [68]	** 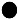 **		** 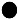 **		** 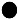 **						** 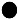 **						** 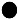 **	** 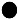 **
	(Evensen et al., 2019) [67]	** 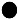 **	** 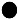 **									** 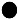 **		** 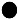 **	** 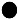 **		** 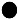 **		
	(Fleiner et al., 2019) [66]							** 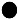 **					** 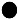 **	** 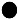 **			** 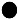 **		
	(Jaiswal et al., 2020) [119]	** 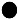 **																** 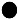 **	
	(Leung et al., 2015) [95]	** 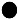 **																** 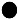 **	
	(Mahlberg et al., 2007) [42]	** 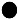 **										** 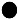 **							
	(Maybrier et al., 2019) [94]	** 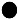 **										** 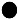 **					** 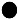 **		
	(Osse et al., 2009) [38]	** 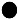 **										** 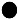 **					** 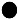 **		** 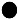 **
	(Stubbs, 2007) [43]										** 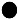 **		** 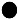 **						
	(Tanev et al., 2017) [65]	** 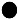 **																** 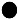 **	
	(Todd et al., 2017) [93]	** 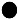 **																** 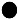 **	
	(Valembois et al., 2015) [33]	** 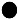 **										** 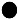 **						** 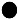 **	
Intensive Care	(Arttawejkul et al., 2020) [48]	** 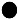 **																** 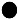 **	
	(Beecroft et al., 2008) [54]	** 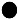 **																** 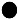 **	
	(Chen et al., 2012) [49]	*		*														** 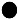 **	
	(Estrup et al., 2019) [64]	** 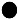 **										** 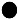 **							
Other Surgery	(Ida et al., 2019) [92]	** 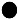 **																** 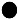 **	
	(Jonsson et al., 2019) [44]				** 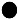 **							** 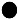 **	** 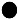 **						
	(Matsuo et al., 2015) [108]				** 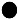 **							** 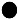 **	** 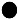 **						
Older Adults	(Beveridge et al., 2015) [116]	** 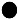 **										** 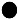 **						** 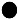 **	
	(Brown et al., 2009) [3]		** 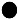 **	** 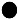 **										** 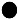 **			** 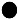 **		
	(Cohen et al., 2019) [45]			** 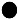 **									** 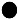 **						
	(Dzierzewski et al., 2014) [115]	** 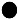 **																** 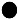 **	
	(Evensen et al., 2017) [101]		** 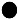 **											** 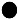 **	** 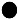 **				
	(Fisher, 2011) [63]			** 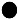 **									** 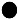 **						
	(Hartley, 2018) [114]		** 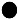 **									** 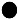 **		** 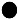 **			** 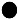 **		
	(Klenk et al., 2019) [107]		** 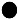 **										** 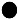 **		** 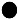 **				
	(Kolk et al., 2021) [62]	** 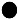 **											** 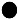 **						
	(Lim et al., 2018) [61]	** 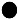 **		** 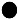 **								** 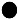 **	** 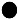 **						
	(McCullagh, 2016) [113]			** 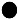 **									** 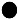 **						
	(Moreno et al., 2019) [46]	** 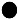 **										** 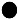 **	** 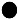 **				** 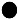 **		
	(Norheim et al., 2017) [106]		** 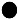 **										** 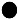 **	** 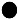 **			** 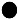 **		
	(Ostir et al., 2013) [10]			** 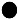 **									** 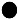 **						
	(Pedersen et al., 2013) [4]		** 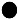 **							** 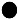 **			** 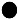 **	** 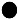 **			** 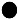 **		
	(Tasheva et al., 2020) [60]	** 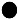 **										** 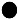 **					** 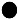 **		
	(Thorup et al., 2017) [53]		** 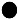 **											** 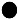 **					
Mixed admissions	(Alessi et al., 2008) [105]	** 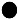 **																** 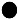 **	
	(Chaboyer et al., 2015) [58]						** 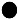 **					** 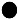 **			** 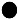 **		** 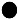 **		
	(Enomoto et al., 2010) [57]				** 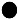 **													** 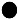 **	
	(Fisher et al., 2016) [9]			** 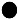 **									** 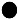 **						
	(Macfarlane et al., 2019) [56]					** 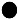 **												** 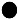 **	
	(Missildine et al., 2010) [55]	** 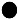 **																** 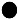 **	
	(Sallis et al., 2015) [112]			** 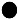 **									** 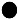 **						
	(Shear et al., 2014) [111]	** 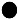 **																** 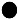 **	
	(Vinzio et al., 2003) [22]	** 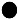 **																	** 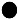 **
Oncology	(Chang et al., 2018) [110]	** 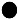 **																	** 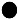 **
	(Fernandes et al., 2006) [109]	** 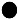 **										** 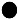 **						** 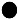 **	** 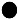 **
	(Jakobsen et al., 2020) [37]	** 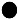 **																** 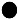 **	
	(Jonker et al., 2020) [91]	** 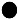 **										** 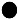 **	** 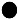 **						
	(Porserud et al., 2019) [47]		** 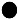 **				** 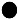 **						** 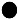 **	** 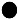 **	** 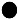 **		** 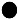 **		
	Morikawa et al. (2018) [90]				** 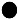 **								** 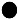 **						
PAR	(Ito et al., 2020) [89]				** 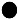 **							** 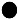 **					** 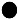 **		

Symbols: 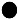 = single sensor, ❷ = bilateral (left and right), * = optional location (either/or), row shaded = both PA and SCR outcomes. Abbreviations: PAR = Parkinson’s disease, PA = Physical activity, SCR = Sleep/circadian rhythm.

**Table 2:** Summary of physical activity domains and metrics.

Activity Domain	Description	Frequency of Papers Assessing Physical Activity Metrics % (n)
Volume Measures	Time spent in physical activity for a specified time frame	97% (65)
Number of steps/step counts	Number of individual steps over a given time period.	48% (32)
Time spent upright	Time spent standing and/or walking.	37% (25)
Time spent lying and/or sitting	Time spent in a lying and/or sitting position	24% (16)
Activity counts per day	An arbitrary term to describe acceleration integrated over a given time period (epoch)	19% (13)
Number of postural transitions	The number of transitions from sitting to standing and/or standing to sitting.	9% (6)
Number or duration of bouts	A bout is defined as the time between two activity events (e.g., an episode of stepping)	7% (5)
Vector magnitude metrics	Derived from raw acceleration data in multiple axes as determined by each study: Euclidean norm minus one (ENMO)/Signal vector magnitude (SVM)/Root mean-squared activity (RMSactivity) (per time period)	7% (5)
Other acceleration metrics	Epoch-related aggregated metric: Motor Activity index (MA_e1_:MA_e2_)/Asymmetry Rate Index(AR_1_24h_:AR_2_24h_)	1% (1)
Intensity Measures	The rate of magnitude in which physical activities are performed, indicating metabolic demand of the activity	24% (16)
Active minutes defined by various metrics	Acceleration thresholds (SVM)/counts (defined by cut points)/(can be categorised into subgroups (i.e., light/moderate)	12% (7)
Moderate intensity (min per day)	Physical activity that burns between 3.0–6.0 (3.0–6.0 METs) times as much energy per minute as when resting	3% (2)
Moderate or above intensity (min per day)	Physical activity that burns more than 3.0 (>3.0 METs) times as much energy per minute as when resting	6% (4)
MET minutes active per day/week	A MET minute being the amount of energy expended during a minute while resting (various cut points used)	4% (3)
Active/Physical energy expenditure (Calories per day)	The amount of energy required to carry out physical functions (breathing, exercising) when active	4% (3)
Low/light/mild intensity (min per day)	Physical activity that burns between 1.5–3.0 (<3.0 METs) times as much energy per minute as when resting	3% (2)
Total energy expenditure (Calories per day)	The amount of energy required to carry out physical functions (breathing, exercising)	3% (2)
Movement intensity	Walking speed m/s^2^	3% (2)
Vigorous intensity (min per day)	Physical activity that burns between 6.0–9.0 (6.0–9.0 METs) times as much energy per minute as you do resting	1% (1)
Very vigorous intensity (min per day)	Physical activity that burns between >9.0 (>9.0 METs) times as much energy per minute as you do resting	1% (1)
Sedentary (min per day)	Physical activity that burns between 0–1.5 (0–1.5 METs) times as much energy per minute as you do resting	1% (1)
Pattern Measures	The distribution of physical activity per day/week	27% (18)
Night-time activity	Time spent in physical activity during night-time hours determined by each study	13% (9)
Daytime activity	Time spent in physical activity during daytime hours determined by each study	10% (7)
Daily variation in physical activity	Similarities/differences for time in physical activity over specified time period e.g., days	7% (5)
Hour to hour variation in physical activity	Similarities/differences for time in physical activity over specified time period e.g., 24 h	6% (4)
Evening activity	Time spent in physical activity during evening hours determined by each study	6% (4)
Morning activity	Time spent in physical activity during morning hours determined by each study	4% (3)
Afternoon activity	Time spent in physical activity during afternoon hours determined by each study	4% (3)
Bout length variation	Similarities or differences in the episodes of activity (e.g., bout lengths) for a specified period of time	1% (1)

ENMO = Euclidean norm minus one, SVM = Signal vector magnitude, MET = Metabolic equivalent of task.

**Table 3:** Summary of sleep and circadian rhythm domains and metrics.

Sleep and Circadian Rhythm Domain	Description	Frequency of Papers Assessing Sleep/Circadian Rhythm Metric % (n)
Sleep Measures	Variables used for study of sleep quality (metrics to define sleep depend on study criteria)	86% (25)
Total sleep time at night	Time spent asleep over the night period. (Depending on the criteria and methodology applied to the study)	76% (22)
Sleep efficiency	Percentage of time between sleep or bedtime and final awakening, which was spent asleep (Depending on the criteria and methodology applied to the study)	48% (14)
Wake after sleep onset (minutes) (WASO)	Amount of time awake during the night after sleep onset to sleep offset (can be displayed as a %)	28% (8)
Total sleep time during the day	Time spent asleep over the day period. (Depending on the criteria and methodology applied to the study)	28% (8)
Number of awakenings	Number of wakeful events after sleep onset (Depending on the criteria and methodology applied to the study).	28% (8)
Sleep-onset latency	Time from the intention to sleep to sleep onset (e.g., lights out/lying in bed, as defined by study)	21% (6)
Total wake time (minutes)	Time spent awake over specified time period, e.g., at night/24 h. (Depending on the criteria and methodology applied to the study)	17% (5)
Total sleep time (TST)	Time spent asleep over specified time period. (Depending on the criteria and methodology applied to the study).	14% (4)
Awake index/Sleep fragmentation index	Number of awakenings divided by the time difference between the initiation of sleep and the offset of sleep	7% (2)
Sleep bouts—number/duration	Sleep bout defined as between two waking events (Depending on the criteria and methodology applied to the study)	3% (1)
Longest sleep episode at night (minutes)	The longest time between two waking events from the onset of sleep	3% (1)
Longest wake episode at night (minutes)	The longest awake time from the onset of sleep	3% (1)
Sleep pattern metrics	Standardised measure for each night’s sleep metric from a set value (usually mean)	3% (1)
Circadian rhythm measures	Variables to study day night variation in rest or rest patterns that follow a 24-h cycle.	21% (6)
Activity amplitude	The difference between the least active 5 h period and the most active 10 h period within each 24-h period	10% (3)
Restlessness Index	Addition of percentage of time spent moving and the percentage immobility phases of 1 min	3% (1)
Interdaily stability (IS)	24-h rhythmic component in evaluating the ‘invariability’ between days	3% (1)
Lowest mean activity during any stretch of 5 continuous hours (L5)	Mean activity of the 5 hr with the lowest activity within the 24 hr	3% (1)
Highest mean activity during any stretch of 10 continuous hours (M10)	Mean activity of the 10 h with the highest activity within the 24 h	3% (1)
Intradaily variability (IV)	Fragmentation of the rhythm (Representing the frequency and extent of transitions between rest and activity)	3% (1)
I < O	A percentage of activity counts that are less than the median activity during the rest span.	3% (1)
24-h autocorrelation coefficient (R24)	Comparing activity data during each 1-min ‘‘epoch’’ of a 24-h period with the activity levels during subsequent epochs	3% (1)
Cosinor parameter: acrophase	Crest time of the fitted rhythmic function, or time of peak activity	3% (1)
Cosinor parameter: amplitude	Half of the difference between the peak and the trough of the rhythm, or half the maximum height of the oscillation	3% (1)
Cosinor parameter: mesor	Rhythm adjusted mean	3% (1)
Cosinor parameter: percent rhythm	Percent of the variance in activity accounted for by the cosine curve	3% (1)

## Data Availability

Data may be shared upon request.

## Supplementary Materials

The following supporting information can be downloaded at: https://www.mdpi.com/article/10.3390/s23104881/s1, Table S1: Search terms used for the review; Table S2: Quality assessment for all included studies; Table S3: The sensor models and versions used to assess physical activity and sleep/circadian rhythm outcomes. Figure S1: The frequency of papers assessing (a) Physical activity metrics (b) Sleep/circadian rhythm metrics.

## Appendix A

**Table A1:** Studies identified in the review for physical activity outcomes.

Study	Study Setting (Admission Type)	Sample Size	Age: Mean [Standard Deviation]	Sensor Technology	Monitoring Duration	Sensor Characteristics/Signal Processing	Reported Measures	Recruitment and Retention	Sensor Removal/Missing Data (Participant Feedback)
Stroke									
Askim et al., 2013 [86]	Observational/Longitudinal: University Hospital/Norway (Emergency)	n = 28	78.7 [8.7]	ActivPAL2, with a tilt switch (1 axis) (Gorman Promed Pty Ltd., Victoria, Australia)	Continuous: Time 1—Hospital, 24 h; Time (2–4), post discharge, 3 days/nights.	Sampling frequency—10 Hz.	Volume: Time spent upright (standing/walking) ‘min’; Number of transitions; Sedentary Behaviour: Time spent lying and sitting ‘min’.	64% retention (recruited to analysed)	Time 1: n = 5 died, n = 11 withdrew, n = 7 missing at random/short recording time: Wrong attachment and instrument failure *.
Gebruers et al., 2013 [84]	Observational/Prospective: Neurology Ward/Belgium (Emergency)	n = 148, Acute stroke = 129/Control = 19	Acute stroke-74.0 [11.4]/Control—71.0 [14.0]	Octagonal basic motion loggers (Ambulatory Monitoring Inc.; Ardsley, NY, USA)	Continuous: 48 h (24 h analysed)	Data recorded in the proportional integrated mode (PIM), epoch (1 s), rebinned into 30 min epochs using a Java program (JBuilder version 3.0)	Volume: Counts; Ratios (activity of impaired arm/activity of nonimpaired arm)	NR	n = 5 accelerometer malfunction
Iacovelli et al., 2019 [83]	Observational/Prospective: Academic Hospital/Stroke Unit: (Emergency, control = Orthopedic disease)	n = 37, Stroke = 20/Control (Orthopedic) = 17	Stroke—69.2 [10.1]/Control—70.4 [4.8]	EZ430-Chronos, Texas Instruments, Dallas, TX, USA	Continuous: 24 h	Acceleration at 33 Hz; Sampling rate (10-bit resolution over a 4 g full scale); epochs (1 min)	Volume: Epoch-related Motor Activity index 1 and index 2 (the standard deviation of the acceleration module and the module of the standard deviation of acceleration components, respectively); 24 h motor performance (mean values over 24 h); Asymmetry Rate Indices to show left or right motor activity prevalence.	NR	NR
Kerr et al., 2016 [82]	Observational/Prospective [2 sites]: Acute Hospital/Medical ward/Scotland: (Emergency)	n = 41	69 [11]	ActivPAL™ (1 axis) (PAL Technologies Ltd., Glasgow, UK)	Continuous: Time 1—hospital, 48 h; Time 2—home, 48 h.	Sampling frequency = 20 Hz	Volume: Time spent standing, stepping (minutes/day); Number of steps taken (per day). Sedentary behaviour: Time spent sitting/lying	21% recruitment (eligible to recruited), 90% retention (recruited to analysed)	n = 2 skin irritation/feeling unwell. n = 1 technical fault
Kunkel et al., 2015 [87]	Observational/Prospective: University Hospital/UK: (Emergency)	n = 74 (at least one activity monitoring session)/Hospital only = 61	At least one monitoring session—76, range = [44 to 95] NR for hospital only	ActivPAL™ physical activity logger (PAL Technologies Ltd., Glasgow, UK)	Discontinuous—Daytime hours	NR	Volume: Walking—steps/minute; Time spent standing or walking; Sedentary behaviour: Time spent sitting/lying	76% retention (recruited to data collected)	n = 9 discharged, n = 7 death, n = 2 withdrawn, n = 1 moved, n = 1 differential diagnosis, n = 4 excluded/malpractice enquiry
Norvang et al., 2018 [81]	Observational/Prospective: University Hospital/Stroke Unit/Norway: (Emergency)	n = 58	75.1 [12.0]	ActivPALs from PAL Technologies Ltd., Glasgow, UK	Continuous: ≥3 days	Sampling frequency = 10 Hz; Battery capacity = 14 days	Volume: Time in upright positions (daily average)/duration of upright bouts/Sedentary behaviour: Time sitting and lying/duration of sitting bouts. (Threshold for transitions—1.5 s). Pattern: Daily variation of average time in lying, sitting, upright positions, and duration of sitting and upright bouts	[unclear]	n = 39 discharge less than 3 days, n = 6 technical error
Sheedy et al., 2020 [80]	Observational/Prospective: Regional tertiary hospital/Stroke unit/Australia: (Emergency)	n = 78, <3 days monitoring = 24/>3 days = 54	Overall median = 80.5, IQR = (70–86), >3 days monitored median = 82.5, IQR = (74–86)	ActivPAL™ (PAL Technologies Ltd.©, Glasgow, UK).	Continuous: ≥3 days up to 14 days (3 days analysed)	*ActivPal proprietary software.*	Volume: Standing time (min), % of time spent standing/stepping, number of steps; Sedentary behaviour: Time (min) spent inactive (lying or sitting)	63% recruitment (screened to recruited), 95% retention (78 wore sensors)	n = 2 not tolerated, n = 2 device failed
Strommen et al., 2014 [79]	Observational/Prospective: University Hospital/Stroke Unit/Denmark: (Emergency—acute ischemic stroke or transient ischemic attack (TIA))	n = 100, Transient ischemic attack (TIA) = 43/Ischemic stroke = 57	TIA—68.9 [10.7]/Stroke—70.2 [13.4]	Actical accelerometers (Philips Respironics)	Continuous: up to 7 days	Epochs of 15 s	Volume: Activity counts (median total per day); Sedentary behaviour: Inactivity-activity count = 0 in all 5 accelerometers during 5-min periods. Pattern: 24-h pattern (Total Raw Activity Counts—from 5 sensors per hour)	81% recruitment (eligible to recruited), 100% retention	2.85% of the total file time is missing data—erroneous recording/non wear time/recording error
Respiratory Condition									
Borges and Carvalho, 2012 [78]	Observational/Prospective: General Hospital/Medical Unit/Brazil: (Emergency—exacerbation of COPD)	n = 20	68.6 [10.7]	DynaPort Moviemonitor (McRoberts, The Netherlands)	Discontinuous: 12 h day, 0800 to 2000; Time 1—(2 days) 3rd and 4th day following admission; Time 2–1 month post discharge	*Manufacturer software*	Volume: Number of steps and total active time (% spent standing, walking, others); Sedentary behaviour: Total inactive time, time spent, sitting and lying	42% recruitment (eligible to recruited), 63% retention (recruited to analysed)	n = 4 early discharge, n = 3 transferred to ICU, died n = 2, n = 2 refused to appear in hospital 1 month after discharge, n = 1 another problem
Dall et al., 2019 [40]	Randomised: University Hospital/Pulmonary Ward/Denmark: (Asthma, Cancer, COPD, Dyspnoea, Pleural effusion, Pneumonia, Pneumothorax, Other)	n = 93, Feedback = 45/Non-feedback = 48	Feedback 73.8 [12.8]/Non-feedback 71.9 [13.6]	Tri axial Accelerometer [no details]	Continuous: ≥24 h	Sampling frequency—12.5 Hz; Epochs of 10 s. (*proprietary software*)	Volume: Average daily time out of bed minutes/day (standing and walking); Sedentary behaviour: Average daily time spent in bed (lying down), sitting and inactive standing. (Visual feedback colour thresholds based on time in body positions)	30% recruitment (eligible to recruited), 66% retention (recruited to analysed)	n = 3 withdrew, n = 1 allergic reaction to the BandAid, n = 25 (feedback group), n = 19 i(no feedback group) recordings of <24 h
Donaire-Gonzalez et al., 2011 [51]	Observational/Prospective/Validation study [8 sites]: Academic Hospital/Spain: (Rehab—COPD)	n = 172	70 [8]	SenseWear Pro 2 Armband (2 axis) (BodyMedia, Inc., Pittsburgh, PA, USA).	Discontinuous: 8 a.m.–10 p.m., ≥3 days to 8 days	NR	Volume: Number of steps per day. Intensity: Time of—any activity (>1.4 METs); Mild activity (>2.5 MET); Moderate activity (>3.6 MET); Energy expenditure during activity >1.4 MET, kcal/day Sedentary behaviour: <30 min/day of moderate activity	NR	NR
Orme et al., 2019 [77]	Observational/Prospective: Teaching Hospital/Cardiorespiratory unit/UK: (Emergency—exacerbation of chronic respiratory disease)	n = 259	70.0 [9.7]	SenseWear Pro 3 Armband (BodyMedia, Inc., Pittsburgh, PA, USA).	Continuous: —up to 14 days	Epochs of 60 s	Volume: Daily step count. Pattern: average step count—overnight, morning, afternoon and evening	NR	n = 7 incomplete data, device malfunction (number not provided)
Pitta et al., 2006 [88]	Observational/Prospective: University hospital/Respiratory ward/Belgium: (Emergency—deterioration in respiratory status)	n = 17	Median = 69, IQR = [60–78]	DynaPort Moviemonitor (McRoberts, The Hague, The Netherlands)	Discontinuous: 12 h days; Time 1—day 2 and day 8 of admission; Time 2—post discharge.	Individual calibration: patient’s body characteristics (height, size of the abdomen).	Volume: Time spent standing, during the day/minutes; Sedentary behaviour: Time spent standing, sitting and lying during the day/minutes Intensity: Movement intensity m/s^2^	71% retention (recruited to analysed)	n = 1 early discharge, n = 3 required intensive care, n = 2 refused to continue
Tsai et al., 2016 [76]	Observational/Prospective: Tertiary hospital/Respiratory ward/Australia: (Emergency—exacerbation of chronic obstructive pulmonary disease)	n = 50	71 [10]	SenseWear1 Armband (SWA; BodyMedia1, Pittsburgh, PA, USA	Continuous: Time 1—Hospital (3 days); Time (2, 3) home post discharge, 7 days;	NR	Volume: Total steps per day. Intensity: Total energy expenditure (calories/day), average (METs) per day, Active energy expenditure (>3.0 METs; calories/day); Physical activity duration (minutes/day) for light (1.5–3.0 METs), moderate (3.0–6.0 METs), vigorous (6.0–9.0 METs) and very vigorous (9.0 METs); Sedentary behaviour: Sedentary (0–1.5 METs)	35% recruitment (screened to recruited), 95% retention (recruited to completion at time point 1)	n = 1 skin irritation, n = 3 dropouts, n = 2 removed sensor
Cardiac Medical/Surgical									
Cook et al., 2013 [39]	Observational/Prospective: Hospital/ICU discharge ward/USA: (Elective—cardiac surgery)	n = 149	67.8 [9], Range = [52–90]	Fitbit (Fitbit, Inc, San Francisco, CA)	NR	Configured to shortest stride length, (*Fitbit website—proprietary software*)	Volume: Steps per day (Median, IQR). Pattern: Step per day variation over each hospital day	NR	n = 2 deaths, n = 1 lost sensor
Floegel et al., 2019 [75]	Observational/Prospective/Pilot [2 sites]: Community hospital/Medical Unit/USA: (Emergency—heart failure)	n = 27	78.0 [9.8]	ActivPAL 3 (PAL Technologies Ltd.©, Glasgow, UK)/Tractivity (Kineteks Corp., Vancouver, BC, Canada)	Continuous: Hospital stay +30 days post discharge	NR	Volume: Time standing, Time ambulating (mean + SD); Hospital steps per 24 h; Sedentary behaviour: Time sitting, time lying % per 24 h	45% recruitment (approached to recruited), 93% retention (recruited to completed)	n = 2 dislodged and removed by patient. Participant feedback: Semi-structured interview—participant themes during their hospital stay were ease of wear and compatibility with hospital technology
Izawa et al., 2015 [103]	Observational/Prospective: University hospital/Medical-Surgical Unit/Japan: (Emergency and Elective—Elderly Cardiac inpatients—myocardial infarction, CABG, valve replacement/heart failure)	n = 268, Female = 75/Male = 193	Male—73.4 [6.2]/Female—73.1 [5.7]	Kenz Lifecorder (1 axis) (Suzuken Co, Ltd., Nagoya, Japan)	Continuous: 4 days (middle 2 days analysed)	Steps and PAEE based on pre-entered age, sex, height, and weight data. *Proprietary software.*	Volume: Total number of steps taken (average). Intensity: Average kcal expended over 2 days [Daily (PAEE) computed by the accelerometer every 4 s, using body weight (W) and a proprietary manufacturer’s factor Ka (exercise index)	47% recruitment (screened to inclusion) Unclear retention—[27 excluded due to incomplete data]	n = 27 data incomplete *
Mungovan et al., 2017 [100]	Observational/Prospective: Private Hospital/Surgical/Australia: (Elective—cardiac surgery)	n = 83, CABG = 36, Valve = 35, CABG and valve = 12	Overall -66 [12], CABG—67 [9]/Valve—63 [14]/CABG and valve—70 [10]	SenseWear Pro 3 Armband (2 axis) (BodyMedia, Inc., Pittsburgh, PA, USA).	Continuous: 5 days	*SenseWear Professional Software* (*version 6.1*)*. Proprietary software*	Volume: Daily step count. Intensity: Physical activity intensity (METs); Duration of exercise >3 METS (min); Duration of exercise less than 3 METS (min)	Unclear recruitment/retention [106 screened, data available for 83]	n = 1 intolerance to the sensor. 90% met minimum compliance
^§^ Redeker and Wykpisz, 1999 [118]	Observational/Prospective: University-affiliated Coronary Care Centre/Acute care/USA: (post coronary artery bypass surgery)	n = 22, middle age = 8, Older adults = 14	Middle age—57.12 [6.62]/Older adults—72.36 [4.14]	The Mini Motion Logger (Ambulatory Monitoring Inc., Ardsley, NY, USA)	Continuous: during admission	Epochs of 60 s. Programmed for zero crossing mode. *Proprietary software*	Volume: Total activity counts. Pattern: Activity counts during 12-h intervals (day 0700–1900 h, night 1900–0700 h Circadian Rhythm: Acrophase (*crest time of the fitted rhythmic function, or time of peak activity*)*;* Amplitude (*half difference between peak and trough of the rhythm, or half maximum height of the oscillation*); Mesor (*rhythm adjusted mean*); Percent rhythm (*% variance in activity*)	NR	NR
Takahashi et al., 2015 [117]	Observational/Prospective: Cardiovascular centre/Japan: (Cardiac surgery)	n = 133	Overall—66.4, range [38–84], Cardiac re-hospitalization—71.6 [5.6]/No Cardiac re-hospitalization—65.7 [9.5]	Active Style Pro HJA-350IT (Omron Healthcare, Kyoto, Japan)	Discontinuous—>8 h/day, during admission; 3 days analysed	NR	Volume: Number of steps walked during last three days of admission (Mean/SD)	83% retention (recruited to analysed)	n = 6 did not wear activity monitor as required
Thorup et al., 2017 [53]	Observational/Prospective/Validation study [2 sites]: University Hospital/Cardiothoracic Surgery and Cardiology Department/Denmark: (Elective and Emergency admission—cardiac disease)	n = 24 (hospital)	Hospitalised—67 [10.03]	The Zip (FITBIT, 405 Howard Street San Francisco, CA 94105, USA)/Shimmer3 (Gyroscope) (Shimmer Research, Dublin, Ireland).	Continuous: Time 1—inpatient, 24 h; Time 2—home 4 weeks later, 24 h	The Zip (*proprietary software*). Shimmer (Sample rate = 50 Hz)	Volume: Total steps per day; % relative error (between sensor models) for step count time periods of 24 h and time periods of 3 min	85% recruitment (approached to recruited) 73% retention (recruited to completion timepoint 1)	n = 1 early discharge, n = 7 withdrew
Orthopedic Surgery/Fractures									
Davenport et al., 2015 [73]	Observational/Prospective: Metropolitan Hospital/Acute orthopaedic ward/Australia: (Emergency—Hip Fracture requiring surgical management)	n = 20	79.1 [9.3]	ActivPAL™ (PAL Technologies Ltd.©, Glasgow, UK).	Discontinuous: 9 a.m.—6 p.m.; Time 1—on admission, 1 day; Time 2—2 weeks later, 1 day.	NR	Volume: Time spent standing and walking (%), Average steps per day. Sedentary Behaviour: Time spent lying/sitting (%);	100% retention [timepoint 1], (93% timepoint 2)	n = 1 device removed <24 h *, n = 1 device malfunctioned
Denkinger et al., 2014 [52]	Observational/Prospective/Validation study: Rehab facility/Geriatric rehab ward/Germany:(Hip fracture)	n = 70	Median = 83, IQR = (79.0–87.3)	Physilog ^®^ (BioAGM, CH)—with single axis gyroscope	Discontinuous: 9 am-6 p.m., 1 day	Sampling rate = 40-Hz	Volume: Time spent walking ‘defined as 3 steps or more’ (min); Time spent upright (min)	NR	n = 2 inter-current illness, n = 2 refusal, n = 1 early discharge
Hayashi et al., 2018 [99]	Observational/Prospective: University Hospital/Japan: (Elective—TKA and THA)	n = 72, TKA = 40/THA = 32	Overall—69.0 [10.5]/TKA—72.4 [7.4]/THA—64.7 [12.4]	Lifecorder GS; Suzuken, Nagoya, Japan	Continuous: 8 days (postop day 3 to 10)	NR	Volume: Total number of daily steps		
Keppler et al., 2020 [72]	Observational/Prospective: University Hospital/Surgical-Trauma Unit/Germany: (Emergency—Orthogeriatric patients with PFF and PHF)	n = 31, PFF = 21, PHF = 10	PFF—80.86 [6.75]/PHF—75.20 [6.86]	Actibelt ^®^, Trium Analysis Online GmbH, Munich	Continuous: Up to 10 days	NR	Volume: Average number of daily steps. Intensity: Walking speed (m/s)	NR	n = 6 dropped out *
^§^ Krenk et al., 2013 [98]	Observational/Prospective: Denmark: Elective—fast track THA and TKA)	n = 20	Overall—70.5, range (61–89)	Actiwatch spectrum ambulatory activity device (Philips Respironics, Murrysville, PA, USA)	Continuous: Time 1, 3 days prior to surgery; Time 2, 7 days postoperatively.	(*Proprietary—Respironics*)	Volume: Maximum activity count per day; Mean activity count per minute; Total activity count (24 h—6 a.m. to 6 a.m.). Sleep: Mean day-time sleep (min); Mean night-time sleep (min); (*Measurement for night-time taken from patients recorded lights-off and lights-on*)	83% recruitment (approached to recruited), 95% retention (recruited to analysed)1 excluded	Sensor never removed for more than 20 min
Kronborg et al., 2016 [71]	Observational/Prospective: University Hospital/Orthopedic ward/Denmark: (Emergency—Hip fracture surgery)	n = 37	80 [8.4]	ActivPAL3™ (PAL Technologies Ltd.©, Glasgow, UK)	Continuous: —Up to 10 days	NR	Volume: Time spent upright ‘min’ (standing and walking); Number of daily upright events (sitting to standing) per 24 h (*Walking defined as an activity in the Z-axis with a cadence of more than 20 steps per minute*)	NR	n = 8 transfer to different unit, isolation, death or discharge, n = 2 technical error
Marsault et al., 2020 [70]	Observational/Prospective: Academic Hospital/Denmark: (Emergency—PFF)	n = 64, Fall hip fracture = 52/fall no fracture = 12	Overall—81.2 [7.8], Hip frac—81.29 [7.45]/Fall group no hip frac—80.83 [9.54]	Axivity™ AX3 tracker (Newcastle upon Tyne, UK)	Continuous: —Time 1, Day 1–3 after operation or admission to the department; Time 2, at discharge and home	Epochs of 60 s; Filter between 0.5 and 20 Hz (wear time analysis); Each minute categorized into “active” or “not active” threshold of SVM > 0.005 (SVM > 0.01 categorized as very high active minutes)	Volume: Signal Vector Magnitude (SVM) (Threshold of SVM > 0.005 for ‘active’ minutes). Intensity: 10-min periods categorized (Sedentary behaviour) 0–10% active minutes, low activity >10–25% active minutes, medium >25–60%, high >60%; Threshold of SVM >0.01 ‘very active’ minutes	97% retention (recruited to analysed)	n = 2 removed
Peiris et al., 2013 [102]	Observational/Prospective: Rehabilitation hospital/Orthopaedic ward/Australia: (lower limb orthopaedic condition, hip or knee replacement, hip fracture)	n = 54	74 [11]	ActivPAL™ (1 axis) (PAL Technologies Ltd.©, Glasgow, UK).	Continuous: 5 days	NR	Volume: steps per day, time spent in upright activities per day (minutes); Time spent walking per day (minutes); Sedentary behaviour: Time spent inactive per day (hours). Intensity: Moderate intensity activity per day (>60 steps/minute), (METs) >3.0, Activity counts > 1075 counts	50% recruitment (screened to recruited), 100% retention	n = 1 redness/minor itching around the dressing that secured the monitor (did not withdraw)
Schmal et al., 2018 [69]	Observational/Prospective: Denmark: (Emergency—post op—PFF)	n = 22	81 [8]	Misfit Shine (Burlingame, CA, USA)/Axivity AX3 (Newcastle upon Tyne, UK)	Continuous: Time 1—24 h, day 2 ± 1 (shortly after operation); Time 2—24 h, 8 ± 3 (shortly before discharge)	Epochs of 60 s; Filter between 0.5 and 20 Hz subjected to a wear time analyses;	Volume: Signal Vector Magnitude (SVM) (Threshold of SVM > 0.005 for ‘active’ minutes). Intensity: Frequency of active minutes grouped as category 1 (“no activity, 0–10%, category 2 (“low activity”) >10–25%, category 3 (“middle activity”) >25–60%, category 4 (“high activity”) >60%; Sedentary behaviour: category 1 (“no activity”)	NR	NR
Twiggs et al., 2018 [96]	Observational/Prospective (Pre-post op): Surgical department/Australia: (Elective—scheduled for TKR)	n = 91, post op ‘in hospital’ = 68	Overall—67.5 [13.1] (NR for post op)	Fitbit Flex	Continuous: Time 1, (2 weeks before op); Time 2, (1 day after op) (analysed days 2–4); Time 3, (6 weeks after op) (7 days for each).	NR	Volume: Daily step counts	72% retention (68 analysed, day 2–4 post-operative period)	Data loss maybe—patient non-compliance, protocol failure with regards to fully charged devices, technical failures of the devices *
van Dijk-Huisman et al., 2020 [41]	Observational/Prospective/Pilot study/Quasi experimental/Interventional: University medical centre/Orthopedic ward/Netherlands: (Elective—post orthopaedic surgery)	n = 97, control group = 64/intervention group = 33; (Analysed overall = 88, control = 61/intervention = 27)	Intervention group (analysed) median = 63.73, IQR = 16.62/Control group (analysed) median = 67.19, IQR = 11.35,	MOX activity monitor (MOX; Maastricht Instruments B.V., Maastricht, The Netherlands./(ADXL362; Analog Devices, Norwood, MA, USA) wireless device: Hospital Fit (HFITAPP0, Maastricht Instruments B.V., Maastricht, The Netherlands).	Continuous: post op to discharge	Sampling frequency = 25 Hz; Range = 8 g; Segmented in to one-second-long windows, fixed non-overlapping sliding window each classified as dynamic or static; Static windows -sensor orientation assessed; Static window—cut-off value of 0.8 g to classify standing or sedentary	Volume: Minutes spent standing and walking per day and per week. Pattern: Variation in minutes spent standing and walking per day. (Days with ≥20 h of wear time were considered valid measurement)	90.7% retention	n = 9 missing data, n = 5 delayed sensor fixation, n = 3 accelerometer malfunctioning
Mixed Admissions (Delirium and Dementia)									
^§^ Davoudi et al. 2019 [68]	Observational/Prospective: University Hospital/ICU/USA: (Emergency—post surgery delirium)	n = 17/Delirious = 4/Non-delirious = 8	Overall Median = 69, IQR = (54.0–73.0)/Delirious Median = 72.5, IQR = (64.5 -74.5)/Non-delirious Median = 62.5, IQR = (37.7–73.0)	Actigraph GT3X (GT3X) devices (ActiGraph, LLC. Pensacola, FL, USA)	Continuous: Up to 7 days	Sampling frequency = 100 Hz; Analysed as 1-min activity counts	Volume: Activity counts (mean/SD); Root Mean Square of Sequential Differences: Root Mean Square of Sequential Differences/Standard Deviation. PATTERN: Activity counts (*daytime 7 a.m.–7 p.m.—night-time 7 p.m.–7 a.m.*) Mean/SD Sleep: Number of immobile minutes (day and night). Circadian Rhythm: M10—Activity intensity of 10-h window with highest sum of activity intensity; L5—Activity intensity 5-h window with lowest sum of activity; Relative amplitude—Difference between M10 and L5.	55% retention (recruited to analysed)	n = 1 or 2 removed at patient’s request, during bathing, or during clinical routines Moved to different ward *
Evensen et al., 2019 [67]	Observational/Cross sectional: University Hospital/Geriatric Ward/Norway: (Emergency–patients with delirium)	n = 60, Hyperactive = 15/hypoactive = 20/Mixed = 17/No-subtype = 8	Hyperactive—86.3 [6.3]/Hypoactive—85.5 [4.4]/Mixed—88.7 [4.7]/No-subtype—86.1 [5.8]	ActivPAL™ (PAL Technologies Ltd., Glasgow, UK)/Actigraph GT3X (GT3X) devices (ActiGraph, LLC. Pensacola, FL, USA)	Continuous: >24 h	Filtered; Epochs into 1 s non-overlapping (using ActiLife software (V.6.13.3)) Threshold of 0.5 (counts s-1) to separate static from dynamic behaviour.	Volume: Activity measured as upright time (minutes) (*upright event minimum 10 s*)*;* Sit-to-stand transitions (numbers); Total wrist activity (counts); Sedentary behaviour: Wrist activity in a sedentary position (%) (*Threshold of 0.5* (*counts s-1*) *to separate static from dynamic behaviour of wrist*)	33% recruitment (eligible to recruited), 58% retention (recruited to analysed)	n = 23 not worn, n = 5 removed sensors, n = 3 worn less than 24 h, n = 5 technical failure, n = 7 sensors lost
Fleiner et al., 2019 [66]	Observational/Cross sectional/Prospective: Academic Hospital/Dementia wards/Germany: (Emergency—Geriatric Psychiatry—Dementia)	n = 87	81 [6.2]	uSense (hybrid motion sensor)	Continuous: 72 h	Software for signal processing and activity recognition: outcome of the FARSEEING EU project	Volume: Time sitting/standing/walking. Daily step counts (mean h/day %); Gait, h/day (%); Sedentary behaviour: Lying, Sedentary sitting/standing, h/day (%)	74% retention (recruited to analysed)	n = 1 refused the sensor attachment, n = 5 removed the device, n = 4 incomplete or missing, n = 1 excluded (used four wheeled walker)
Mahlberg et al., 2007 [42]	Observational/Prospective/Pilot/Interventional: Geriatric Psychiatry Dep/Germany: (probable dementia of the Alzheimer type)	n = 20, Rivastigmine = 10/Placebo = 10	Overall—80.4 [9.1], Rivastigmine—82.6 [7.2]/Placebo—78.2 [10.3]	Actiwatch, Cambridge Neurotechnology Co., Cambridge, UK	Continuous: 2 weeks (First 3 days and last 3 days analysed)	NR	Volume: Activity counts 10^3^. Pattern: Activity counts (diurnal 6 a.m.–9 p.m., nocturnal 9 p.m.–6 a.m., evening 3 p.m.–9 p.m.)	95% retention (recruited to completed)	n = 1 withdrew, n = 2 technical reasons
Maybrier et al., 2019 [94]	Observational/Prospective: Academic Hospital/USA: (Elective-surgical—post operative delirium)	n = 83, no delirium postoperative days (POD) 0–5 = 51/delirium during POD 0–1 = 24/delirium during POD 2–5 = 13 [overlap of subjects for outcome groups]	Intact POD 0–5 Median = 68, IQR = 10/Delirium POD 0–1 Median = 72 IQR = 16/Delirium POD 2–5 Median = 69, IQR = 14	ASPW wActiSleep Plus, ASPB, and wGT3XBT (ActiGraph Corp., Pensacola, FL, USA).	Continuous: 24 h after surgery up to discharge	Sampling frequency = 30 Hz; Counts binned in 1-min intervals across three accelerometer axes;	Volume: Root mean-squared activity (RMSactivity); Median activity counts (MAC); Sedentary behaviour: Number of immobile minutes (NOIM), *defined as—total number of minutes with an RMSactivity count of zero*. Pattern: MACDay; MACNight; MACDay-Night *activity between day and night*	55% retention (recruited to analysed), 83 patients data analysed—due to exclusion	n = 6 device error, n = 32 incomplete activity data during 16:00–6:00
^§^ Osse et al., 2009 [38]	Observational/Prospective: Medical centre/Cardiothoracic surgery dept/Holland: (Elective—Postcardiotomy delirium)	n = 79, ‘Non-cr-Del’(non-clinically relevant delirium) = 46, ‘Short-Del’ = 16, ’Sustained-Del’ = 17	‘Non-cr-Del—72.9 [4.9]/Short-Del—75.2 [4.1]/Sustained Del—75.2 [4.5]	Actiwatch (Cambridge Neurotechnology Ltd., Cambridge, UK)	Continuous: post-surgery for up to 6 days	Epochs of 60 s	Volume: Activity per minute (mean); Sedentary behaviour: Number of minutes immobile (*based on the total number of minutes with a score of zero*). Pattern: Sedentary behaviour: number of minutes Immobile (night-time 23.00–06.00 h and daytime 06.00 h–23.00 h) Circadian Rhythm: Restlessness Index (addition of the percentage of time spent moving and the percentage immobility phases of 1 min); Activity Amplitude (difference between the least active 5 h period and the most active 10 h period within each 24-h period)	90% recruitment (eligible to recruited), 91% retention (recruited to analysed)	n = 7 technical problems
Stubbs et al., 2007 [43]	Observational/Prospective/Intervention (pre-post): Independent psychiatric hospital/Medical psychiatric ward/UK: (Older psychiatric inpatients)	n = 53	71.6, range [51–85]	Pedometer *	Discontinuous: 8.30 a.m.–5.30 p.m., 3 days	NR	Volume: Step count per day (mean)	89% retention (recruited to completion)	n = 3 discharged, n = 2 refused to wear
^§^ Valembois et al., 2015 [33]	Observational/Prospective/Cross sectional: Intermediate care unit/Geriatric ward/France: (Emergency—Dementia and apathy or aberrant motor behaviour)	n = 183	84.9 [6.8]	Vivago (Vivago^®^, Vivago Oy, Espoo, Finland)	Continuous: 10 days	Proprietary software (Vista, Vivago, Finland)	Pattern: Mean motor activity (grouped by pre-defined 3-h periods during the day 0:00 to 2:59, 3.00 to 05.59, 06.00 to 8.59, 9.00 to 11.59, 12.00 to 14.59; 15.00 to 17.59; 18.00 to 20.59 and 21.00 to 23.59) Sleep: Total sleep time (min); Number night awakenings (n); (Proprietary software)	100% retention (obtained analysable actigraphy data for all the patients included)	impaired sensor contact with skin/long period of time out of the detection zone *
Patients In Intensive Care									
Estrup et al., 2019 [64]	Observational/Prospective: University Hospital/ICU/Denmark: (Emergency)	n = 44	72 [10]	Micro SleepWatch^®^ (Ambulatory Monitoring, Ardsley, NY, USA)	Continuous: up to 7 days	*Proprietary software*—activity as a count of movements per time unit in zero crossing mode	Volume: Activity counts per day (mean); Maximum activity in 1 h interval on day 2. Pattern: Mean activity per hour during daytime (7 a.m.–4 p.m.); Mean difference between day and night	67% recruitment (screened to recruited), 93% retention (recruited to analysed).	n = 2 transferred, n = 1 died, non-wear time (readings <50 counts) number not defined
Other Surgery									
Jonsson et al., 2019 [44]	Randomised control trial: University Hospital/Surgical/Sweden: (Elective—thoracic surgery—confirmed or suspected lung cancer)	n = 94, intervention = 50/Control = 44	Intervention—69 [8]/Control—68 [8]	ActiGraph, model GT3X+, Manufacturing Technology Inc., Pensacola, FL, USA	Continuous: hospital ≥3 days	Sample frequency = 30 Hz; Epochs of 10 s	Volume: Average counts; Steps per hour	81% recruitment (eligible to recruited—intervention: control, 54:53), 71% retention (recruited to analysed 50:44)	n = 10 early discharge, n = 3 accelerometer malfunction
Matsuo et al., 2015 [108]	Observational/Prospective: Rehab Unit/Japan: (Rehab—lower extremity bypass surgery)	n = 13, active group = 6/inactive group = 7	72.8 [5.9]	Active Style Pro. HJA-350IT, Omron Healthcare	Continuous: 2 days prior to surgery to discharge (except on the day of surgery)	*BI-LINK-analysis software* (Omron Healthcare)	Volume: Daily steps; Maximum walking distance. Intensity: METs-hours/day (*calculated using BI-LINK-analysis software* (*Omron Healthcare*)	NR	NR
Older Adults									
^§^ Beveridge et al., 2015 [116]	Observational/Prospective: Medical Centre/Medical Ward/USA	n = 120 Subgroup activity (with sleep data) (Overall = 300)	Subgroup activity (with sleep data)—65.5 [11.1]	Actiwatch2, Respironics, Inc., Murrysville, PA, USA	Continuous: during admission	*Proprietary software* (Actiware 5 ‘respironics inc’—summed over 15 and 60 s intervals	Volume: Average and maximum Activity Counts (min); Total Activity Counts. (Calculated from reported wake up on morning to bed that night) Sleep: Total time spent asleep (night-time); Sleep efficiency. (*Assumed sleep period based on reported bedtimes and morning wake times*)	42% recruitment (consented to larger study to recruited), 71% retention rate [recruited to analysed]	n = 104 removed sensor at night or sensor failure, n = 49 removed during the day,
Brown et al., 2009 [3]	Observational/Prospective: Veterans Affairs Medical Centre/Medical ward/USA	n = 45	74.0 [6.5]	AugmenTech, Inc; Pittsburgh, Pennsylvania] * Only 1 axis used	Continuous: Up to 7 days	Epochs of 20 s	Volume: Mean % of 1 hr intervals each day spent, standing/walking. Sedentary behaviour: Mean % of 1 hr intervals each day spent lying + sitting	90% retention (recruited to analysed)	n = 2 declined health, n = 2 withdrew, lack of data within 48 h of admission or having less than 23 h of data *
Cohen et al., 2019 [45]	Quasi-experimental/Interventional/pre-post: Medical centre/Internal medical unit/Northern Israel	n = 377, Control = 188/Intervention group = 189	75.1 [7]	Actical accelerometers (Philips Respironics)	Continuous: Up to 3 days	NR	Volume: Mean number of steps per day.	45% recruitment (approached to recruited), 94% retention (recruited to completion)	n = 6 died, n = 3 became delirious, n = 9 withdrew, n = 1 transferred, n = 1 deterioration, n = 8 early discharge,
Evensen et al., 2017 [101]	Observational/Prospective: Academic Hospital/Geriatric Ward/Norway (90% emergency)	n = 38	82.9 [6], Range = [67.6–92.5]	ActivPAL™ (PAL Technologies Ltd., Glasgow, UK)	Continuous: During admission, 24 h analysed	NR	Volume: Number of upright events (*minimum event 9.9 s*); Lengths of upright events (minutes); Maximum length of upright events (minutes); Upright event variability (IQR). Pattern: time in upright position night (00–06), morning (06–12), afternoon (12–18), evening (18–24)	88% retention (recruited to analysed)	n = 5 missing data *
Fisher et al., 2011 [63]	Observational/Prospective: Teaching Hospital/Acute care for elderly/USA (Emergency)	n = 239	76 [6], Range [65–100]	StepWatch Activity Monitor (SAM) (2 axis) (Modus health, Washington, DC, USA)	Continuous: ≥24 h up to discharge	NR	Volume: Total number of steps per day; Mean daily steps; Total minutes of ambulatory steps (*defined as stride counts recorded by the monitor times two*); Total minutes of ambulatory activity (*number of 1-min intervals recorded by the monitor with a stride count greater than 0*)	74% retention (recruited to analysed)	n = 28 removed *, n = 36 < 1 complete 24-h day (midnight to midnight), n = 18 medical reasons/tests, n = 84 incomplete data *, n = 2 wore SAM home data lost
Hartley et al., 2018 [114]	Observational/Prospective/Feasibility:University Hospital/Department of medicine for the Elderly wards/UK:	n = 24, Men = 12/Women = 12	Overall Median = 80.5, Range = (70.0–95.0), Male Median = 79.5, Range = (70–95])/Female Median = 81.5, Range = (71–89)	Axivity™ AX3 tracker (Newcastle upon Tyne, UK)	Continuous: 48 h	Sampling frequency = 100 Hertz; Range of ± 4 g; Autocalibrated to local gravity; Low-pass filter at 20 Hertz; Epochs of 5 s (summarised from three signals).	Volume: Euclidean Norm Minus One (ENMO)/Time spent moving upright/sitting/standing/lying (%); (*cutoff threshold ENMO value of >13 milligravity units* (*mg*) *for moving*) Sedentary behaviour: Time spent sitting and lying (%)	79% retention (recruited to analysed)	n = 1 sensor fell off, n = 4 early discharge, n = 1 removed for MRI scan. Participant feedback: Questionnaire–All participants report ‘no discomfort’, with sleep not affected.
Klenk et al., 2019 [107]	Observational/Prospective: Geriatric Hospital/Germany: (Rehab)	n = 647	82.0 [7.19]	ActivPAL3™ (PAL Technologies Ltd.©, Glasgow, UK)	Continuous: day 2 and day 15 of admission (24 h x2)	NR	Volume: Mean walking duration; Walking bout duration (*interval between two periods of standing*); Number of sit-to-stand transfers	52% retention (recruited to analysed)	n = 555 missing data on day 2 or 15, n = 49 incomplete 24 h *
Kolk et al., 2021 [62]	Observational/Prospective/[6 sites]: General Hospital/Internal;cardiology;geriatric wards/Holland: (Emergency)	n = 188	79.1 [6.7]	Fitbit Flex activity tracker (Fitbit, Inc., San Francisco, CA, USA)	Continuous: hospital admission to 1 week post discharge.	NR	Volume: number of steps per day	Unclear	n = 12 medical, n = 11 died, n = 46 technical/logistic reason, n = 17 lost to follow-up, n = 72 unknown
Lim et al., 2018 [61]	Observational/Prospective/Cross sectional:Acute medical ward/England (Emergency)	n = 38, Men = 18/Women = 20	Overall—87.8 [4.8], Men—88.3 [5.1]/Women—87.5 [4.5]	StepWatch Activity Monitor (SAM) (2 axis) (Modus health, Washington, US) and GENEActiv (Activinsights, Kimbolton, UK)	Continuous: ≥24 h, up to 7 days	Frequency = 100 Hz (GENEActiv)	Volume: Steps per day; Total minutes per day stepping; Minutes spent in different bout lengths Intensity: Minutes per day with acceleration ≥12 milli-g. Pattern: Mean step count and mean acceleration per hour of each day; Minutes in sustained ambulation ≥4 step (1–5 min, 6–10 min, 10+); Minutes spent in different bout lengths ≥ 12 milli-g (1–5 min, 6–10 min, 10+)	NR	Participant feedback: Questionnaire—Acceptability of both devices was high overall, wrist-worn device (96%) was more acceptable to patients than the ankle-worn device (83%)
McCullagh et al., 2016 [113]	Observational/Cross sectional/Prospective: Teaching Hospital/Ireland: (Medical patients)	n = 154	77.5 [7.4]	Stepwatch Activity Monitor (SAM) (Modus health, Washington, US)	Continuous: up to 7 days	Sampling frequency = 128 Hz; Manufacturers software—based on answers to direct questions relating to (height, gait pattern and gait cycle); Epochs of 15 s	Volume: average daily step-count	68% recruitment (eligible to recruited), 97% retention (recruited to analysed)	n = 4 saved incorrectly/irretrievable
Moreno et al., 2019 [46]	Randomised controlled trial: University Hospital/Respiratory and Clinical Medicine/Brazil: (Emergency)	n = 68, Experimental = 33/Control = 35	Experimental—69 [7]/Control—69 [7]	Actigraph GT3X LLC. Pensacola, FL, USA	Continuous: during admission up to 20 days	NR	Volume: Mean steps per day: Sedentary behaviour: Time sedentary (%). Intensity: % of time light, moderate, hard, and very hard intensity level (thresholds ≤ 1951, 1952–5724, 5725–9498, ≥9499 cnts·min-1, respectively	72% recruitment (eligible to recruited), 97% retention (recruited to analysed)	n = 2 died, n = 2 device malfunction
Norheim et al., 2017 [106]	Observational/Prospective: Geriatric Ward/Denmark: (Rehab)	n = 16 (pre-test to post-test)	84.8 [1.9]	ActivPAL™ (PAL Technologies Ltd.©, Glasgow, UK)	Continuous: ≥24 h for both pre-test and post-test (leg resistance exercise)	NR	Volume: Time spent standing, and walking. Sedentary behaviour: Time spent sedentary (lying or sitting),	3% recruitment (screened to consented), 79% retention (recruited to analysed)	n = 3 transferred, n = 1 sensor lost
Ostir et al., 2013 [10]	Observational/Prospective: University Hospital/Acute Care for Elders (ACE) hospital unit/USA: (Emergency)	n = 224	Age—65–74 median = 108, IQR = 48.2/Age -75–84 median = 86, IQR = 38.4/Age—80 median = 30, IQR = 13.4	StepWatch Activity Monitor (SAM) (2 axis) (Modus health, Washington, DC, USA)	Continuous: 24 h at start of admission, 24 h before discharge	NR	Volume: Total steps; Total minutes active (*number of 1 min intervals recorded in a 24-h period with a step count greater than 0*)	87% recruitment (eligible to recruited)), 79% retention (recruited to final sample)	n = 24 removed for medical procedures/tests
Pedersen et al., 2013 [4]	Observational/Prospective: University Hospital/Acute medical admission ward: (Emergency)	n = 48, Ambulatory = 42/Nonambulatory = 6	Ambulatory median = 84.7, IQR (78.6–87.2)/Nonambulatory median = 82.8, IQR (79.9–88.0)	Augmentech, Inc.; Pittsburgh, PA, USA	Continuous: within 48 h of admission up to discharge, <10 days	NR	Volume: Hours per day spent standing and/or walking, Sedentary behaviour: Hours per day spent lying, sitting: To define postures—algorithm identification from two axes by Angle = tan − 1(AX/AZ)	72% recruitment (eligible to recruited), Retention unclear [one excluded due to lack of data]	n = 1 episode of acute psychosis (3 day pause), n = 2 removed after 3 and 4 days, n = 1 lack of accelerometer data * (removals by patients or staff for examinations *)
Tasheva et al., 2020 [60]	Observational/Prospective: University hospital/Medicine ward/Switzerland: (Emergency)	n = 177, Male = 106/Women = 71	Men—79.7 [8.1]/Women—83.5 [8.6]	GENEActiv Original, ActivInsights Ltd., UK	Continuous:—during admission ≥24 h	Sampling frequency = 50 Hz	Intensity: Time spent in light or moderate activity levels (min per day, % of daily time); Sedentary behaviour: *<30 mg inactivity,* (*30–99 mg light, ≥100 for moderate PA*) Pattern: Activity per hour (average) (Physically active—in the highest quartile of time spent in light and moderate PA or spending ≥20 min/day in moderate PA)	56% recruitment (screened to recruited), 84% retention (recruited to analysed)	Very short (<24 h) recordings or had a high percentage of accelerometer nonwear time *
Theou et al., 2019	Observational/Prospective: Tertiary care hospital/Medicine Unit and Geriatric Assessment Unit/Canada: (Emergency)	n = 111, bedridden = 32/person assistance = 44/independent = 35	Overall—82.2 [8.0], bedridden—84.8 [8.5]/person assist—81.6 [6.5]/independent—80 [8.9]	ActivPAL3™ (PAL Technologies Ltd.©, Glasgow, UK)	Continuous: up to 2 weeks	NR	Volume: Upright time (min); Number of upright bouts (per day). Pattern: Upright time and upright bouts during awake (7 a.m.–10 p.m.), night-time (10 p.m.–7 a.m.), morning (7 a.m.–12 p.m.), afternoon (12 p.m.–5 p.m.), evening (5 p.m.–10 p.m.). (*outcomes measured every 15 s*)	45% recruitment (eligible to recruited), 85% retention (recruited to analysed)	n = 11 no valid ActivPAL data, n = 5 no mobility assessment within 48 hr of hospital admission, n = 3 withdrew from study
Mixed Admission									
Chaboyer et al., 2015 [58]	Observational/Prospective: Tertiary Hospital/Acute Medical Wards/Australia: (Emergency)	n = 84, 66 to 74 years = 18/>75 years = 44	Overall median = 77, IQR = [64.0–85.8], 66 to 74 years median = 70, IQR = [68.8–72]/>75 years median = 85, IQR = [81.0–87.0]	ActiGraph, model GT3X+, Manufacturing Technology Inc., Pensacola, FL, USA	Continuous: 24 h	Butterworth low-pass filter with a cut-off frequency of 0.1 Hz;	Volume: Number of postural changes (24 h) (*Angle data for postural change*)*;* Sedentary behaviour: % time sedentary (<100 counts/min); Intensity: % time in light (100–760 counts/min), moderate or greater (>760 counts/min)	NR	NR
Fisher et al., 2016 [9]	Observational/Prospective: Teaching hospital/Acute Medical Ward/USA: (Emergency—cardiovascular, pulmonary, infection, gastrointestinal, or endocrine)	n = 164	76.2 [7.0]	StepWatch Activity Monitor (2 axis) Orthocare Innovations	Continuous: Up to discharge	NR	Volume: Steps per day (mean) (*recorded in 1-min intervals per 24-h day, off-axis accelerations are not registered* i.e., *lying down*)	82% retention (recruited to analysed)	n = 35 either discharged <48 h, withdrew, removed sensor nor resecured.
Sallis et al., 2015 [112]	Observational/Prospective: Community hospital/Medical-surgical units/USA: (ambulatory medical and surgical adult patients)	n = 777, Age 18–40 years = 111/41–65 years = 325/65–75 years = 187/>75 years = 151	Overall—60 [17] (not provided for the subgroup 65 or over)	Tractivity^®^ activity monitor (Tractivity^®^, Vancouver, BC, Canada)	Continuous: during admission	NR	Volume: Median step count per 24 h. Pattern: Distribution of step counts by percentage of accrued steps each hour over 24 h	NR	n = 10 device failure, 44% of sensors were lost mainly as nursing staff failed to remove on discharge, potential non wear by patient *
Oncology									
^§^ Fernandes et al., 2006 [109]	Observational/Prospective/Two group comparative study: Tertiary hospital/UK: (women with cancer and matched median age group)	n = 50, patients = 25/volunteers = 25	Subject median = 67, range = [46–90]/volunteers’ median = 63, range = [54–79]	Actimeters (Ambulatory Monitoring Inc., New York, NY, USA)	Continuous: 72 h	Epochs of 60 s	Volume: Mean activity score (counts/minute) up interval—during the time awake. Sleep: % Sleep—up interval and down interval; Sleep efficiency (*% time asleep when attempting to sleep*)*;* Sleep latency (*minutes trying to sleep until first 20 min block of sleep*)*;* Wake after sleep onset (min); (*Sleep diary used to define up and down interval*) Circadian Rhythm: 24-h autocorrelation coefficient (R24) (*activity data during each 1-min ‘‘epoch’’ of a 24-h period with the activity levels during subsequent epochs*)	100% recruitment rate	NR
Jonker et al., 2020 [91]	Observational/Prospective: Tertiary academic hospital/Surgical Unit/Netherlands: (Elective—Cancer patients scheduled for surgery)	n = 37/Recovery = 15/Not recovered = 22	Recovery—71.7 [4.8]/Not recovered—73.6 [5.2]	Fitbit Charge 2 (Fitbit Inc., San Francisco, CA, USA)	Continuous: Time 1—pre-op 7 days, Time 2—during admission—2 days, Time 3—3 month later 7 days.	NR	Volume: Daily step count. Intensity: Time engaged in moderate-vigorous physical activity (MVPA) (*minutes per day spent on activities with an intensity of 3 Metabolic Equivalent of Tasks*)	49% recruitment (eligible to recruited), 80% retention (recruited to analysed)	n = 1 died, n = 1 dermatitis, n = 1 too stressful, n = 3 surgery cancelled, n = 2 withdrew due to complications, n = 1 too time consuming, n = 1 no data available
Porserud et al., 2019 [47]	Non-randomised controlled trial: University hospital/Sweden: Elective—abdominal surgery—colo-rectal, urinary, ovarian cancer)	n = 133, Activity board = 67/Standard treatment = 66	Overall—68.1 [12.3], Activity board—69.3 [11.4]/Standard treatment—66 [67.0]	activPAL3 micro (PAL Technologies Ltd., Glasgow, UK)	Continuous: up to 5 days	NR	Volume: Time spent standing, stepping, sitting (min/day); Number of step counts; Sit to stand transitions. Sedentary behaviour: lying in bed/sitting, min/day (*valid day—12 h wear time*)	89% retention (recruited to analysed)	Less than 12 h of wear time on any of the three days, early discharge *
Morikawa et al., 2018 [90]	Observational/Prospective: Hospital/Oncology Unit/Japan: (Elective—Non small cell lung cancer (Advanced) Elderly—scheduled to initiate first line chemotherapy	n = 18, cachexia = 11/noncachexia = 7	Overall median = 74.5, range [70–82], Cachexia median = 74, Range [70–82]/Noncachexia median = 76, Range = [70–81]	Lifecorder^®^, Suzuken Co., Ltd., Japan)	Discontinuous: daytime, before admission through to during admission, 1st , 2nd, and 3rd week after discharge	NR	Volume: Daily steps (mean).	96% recruitment (screened to recruited), 72% retention rate (recruited to completion)	n = 1 failure of accelerometer, (less than 5 h a day then that day excluded *
Parkinsons Disease									
Ito et al., 2020 [89]	Observational/Prospective/Pilot study: Medical Centre/Neurology Department/Japan: (Elective—admitted for adjustment of medication or deep brain stimulation)	n = 11	67.1 [7.7]	Active Style Pro HJA-350IT, Omron Healthcare, Kyoto, Japan	Discontinuous: 10 h per days 07:00/07:30 am—05:30/07:00 p.m., during admission	Sampling frequency = 32 Hz; Range = 23.0 g; Epochs of 10 s	Volume: Time spent in physical activity (active/inactive); Sedentary behaviour: Time spent sedentary and inactive; Intensity: Physical activity level calculated by Total Energy Expenditure derived from a manufactured regression equation using METs assessed by the triaxial accelerometer. Pattern: METs variation over a day		

Symbols: * No further information provided, **^§^** author/shaded row study identified as reporting both physical activity and sleep/circadian rhythm outcomes. Abbreviations in alphabetical order: CABG = Coronary Artery Bypass Grafting, COPD = Chronic Obstructive Pulmonary Disease, IQR = interquartile range, L5—least active 5 h of the day, MET = Metabolic Equivalent of Task, M10 = most active 10 h of the day, NR = Not recorded, PAEE—Physical Activity Energy Expenditure, SAVR = Surgical Aortic Valve Replacement, SD = Standard deviation, TAVI = Transcatheter Aortic Valve Implantation, THA = Total Hip Arthroplasty, TKA = Total Knee Arthroplasty, TKR = Total Knee Replacement, PFF = Proximal Femur Fracture, PHF = Proximal Humerus Fractures.

**Table A2:** Studies identified in the review for sleep/circadian rhythm outcomes.

Study	Study Setting (Admission Type)	Sample Size	Age: mean [Standard Deviation]	Sensor Technology	Monitoring Duration	Sensor Characteristics/Signal Processing	Reported Measures	Recruitment and Retention	Sensor Removal/Missing Data (Participant Feedback)
Stroke									
Bakken et al., 2012) [85]	Observational/Prospective [2 sites]: Hospital Trust, University Hospital/Medical Wards/Norway: (Emergency—First time stroke)	n = 90	68.4 [13.3]	Motion logger (Ambulatory Monitoring Inc.; Ardsley, NY, USA)	Continuous: 3 nights/two days	Epochs of 60 s: (*Activity counts analysed in Hertz with the Cole–Kripke algorithm ActionW software* (*Ambulatory Monitoring*)	Sleep: Total sleep time at night (mean minutes); Wake after sleep onset %; Number of awakenings; Daytime nap (mean minutes of sleep between 09:00–20:59). (*Sleep period between 21:00–08:59*)	65% recruitment (screened to recruited) 95% retention (Time 1)	n = 3 deaths, n = 3 transfers, n = 3 missing data *, n = 13 not completed protocol/assessments.
Cardiac Medical/Surgical									
Amofah et al., 2016 [104]	Observational/Prospective: University Hospital/Cardiothoracic Surgical Centre/Norway: (Emergency and Elective -Octogenarians undergoing SAVR and TAVI	n = 143, SAVR = 78/TAVI = 65	Overall—83 [2.7], SAVR—82 [2.0]/TAVI—85 [2.8]	Actiwatch 2 (Respironics, Philips Health Care, Best, The Netherlands	Continuous: 5 days post op	NR	Sleep: Total sleep time at night; Sleep efficiency; Wake time at night; Sleep time during day (*between 07:00 and 23:00*). (*Sleep period between 23:00 and 07:00*)	89% recruitment (eligible to recruited) Unclear retention (7 nonresponsive/died *)	n = 7 non-responsive due to sedation, or died within five days after surgery
Gimenez et al., [50]	Observational/Prospective/Interventional: University Hospital/Cardiology Ward/Netherlands	n = 196, Intervention (dynamic light/dark cycle) = 100/Control = 96	Overall—66.5 [13.1], Intervention—68.1 [12.2]/Control—64.9 [13.9]	Actigraph [no model provided]	Continuous: during admission	NR	Sleep: Total sleep duration (min); Sleep-onset latency (*Measurement for time to bed/time in bed not clear*)	34% recruitment, 47% retention	n = 27 withdrew, n = 20 transferred, n = 5 moved to other room, n = 22 early discharge (<2 days, n = 2 unknown, n = 1 still hospitalised, n = 1 migraine, n = 1 skin irritation,
Redeker and Wykpisz, 1999 [118]	Observational/Prospective: University-affiliated Coronary Care Centre/Acute care/USA: (post coronary artery bypass surgery)	n = 22, middle age = 8, Older adults = 14	Middle age—57.12 [6.62]/Older adults—72.36 [4.14]	The Mini Motion Logger (Ambulatory Monitoring Inc., Ardsley, NY, USA)	Continuous: during admission	Epochs of 60 s. Programmed for zero crossing mode. *Proprietary software*	Volume: Total activity counts. Pattern: Activity counts during 12-h intervals (day 0700–1900 h, night 1900–0700 h Circadian Rhythm: Acrophase (*crest time of the fitted rhythmic function, or time of peak activity*)*;* Amplitude (*half difference between peak and trough of the rhythm, or half maximum height of the oscillation*); Mesor (*rhythm adjusted mean*); Percent rhythm (*% variance in activity*)	NR	NR
Takaesu et al., 2015 [74]	Observational/Prospective: Medical University Hospital/Coronary Care Unit/Japan: (Emergency)	n = 42, Subjects = 23/Control = 19	NR	Actigraph [no model provided]	Continuous: 24 h, 6 a.m.—6 a.m.	NR	Sleep: Total sleep time at night (min); Total sleep time in the day (min); Sleep latency (min); Wake time after sleep onset (min); Sleep efficiency (%) (*diurnal period 9 p.m.–6 a.m., nocturnal period 9 p.m.–6 am*)	100% retention	NR
Orthopedic Surgery/Fractures									
^§^ Krenk et al., 2013 [98]	Observational/Prospective: Denmark: Elective—fast track THA and TKA)	n = 20	Overall—70.5, range (61–89)	Actiwatch spectrum ambulatory activity device (Philips Respironics, Murrysville, PA, USA)	Continuous: Time 1, 3 days prior to surgery; Time 2, 7 days postoperatively.	(*Proprietary—Respironics*)	Volume: Maximum activity count per day; Mean activity count per minute; Total activity count (24 h—6 a.m. to 6 a.m.). Sleep: Mean day-time sleep (min); Mean night-time sleep (min); (*Measurement for night-time taken from patients recorded lights-off and lights-on*)	83% recruitment (approached to recruited), 95% retention (recruited to analysed)1 excluded	Sensor never removed for more than 20 min
Miller et al., 2015 [97]	Observational/Prospective: Tertiary Hospital/Surgical Ward/USA: (Elective—Post-op—THA and TKA)	n = 50	65 [10.8]	Actiwatch 2 (Philips Respironics, Andover, MA, USA)	Continuous: first 2 nights post surgery	Epochs of 15 s: (*Further processing: Actiwatch Respironics software ‘Philips Respironics’*)	Sleep: Total sleep time (night-time); Sleep efficiency (*Sleep diary used*); Awake index (*number of awakenings divided by the time difference between the initiation of sleep and the offset of sleep*)	100% retention rate	NR
Mixed Admissions (Delirium and Dementia)									
^§^ Davoudi et al. 2019 [68]	Observational/Prospective: University Hospital/ICU/USA: (Emergency—post surgery delirium)	n = 17/Delirious = 4/Non-delirious = 8	Overall Median = 69, IQR = (54.0–73.0)/Delirious Median = 72.5, IQR = (64.5 -74.5)/Non-delirious Median = 62.5, IQR = (37.7–73.0)	Actigraph GT3X (GT3X) devices (ActiGraph, LLC. Pensacola, FL, USA)	Continuous: Up to 7 days	Sampling frequency = 100 Hz; Analysed as 1-min activity counts	Volume: Activity counts (mean/SD); Root Mean Square of Sequential Differences: Root Mean Square of Sequential Differences/Standard Deviation. PATTERN: Activity counts (*daytime 7 a.m.–7 p.m.—night-time 7 p.m.–7 a.m.*) Mean/SD Sleep: Number of immobile minutes (day and night). Circadian Rhythm: M10—Activity intensity of 10-h window with highest sum of activity intensity; L5—Activity intensity 5-h window with lowest sum of activity; Relative amplitude—Difference between M10 and L5.	55% retention (recruited to analysed)	n = 1 or 2 removed at patient’s request, during bathing, or during clinical routines Moved to different ward *
Jaiswal et al., 2020 [119]	Retrospective/Observational (secondary analysis of a Randomised Controlled Trial): Teaching Hospital/Medicine Wards/USA: (Inpatient delirium)	n = 70, Delirium = 17/no delirium = 53	81.5 [7.4]	Actiwatch Spectrum Plus (Philips Respironics, Murrysville, PA, USA)	Continuous: during admission	Epochs of 15 s: *Further processing—Actiwatch Respironics software* (*Philips Respironics*)	Sleep: Total sleep time Average 24-h total sleep time; Night-time sleep duration; Number of sleep bouts at night-time; Duration of sleep bout; Wake after sleep onset; Sleep efficiency; Fragmentation Index (using average length of sleep bout during night sleep); (*Rest interval—manual based on decreased activity and light*)	NR [secondary analysis on the complete sensor data]	Secondary analysis of those wearing sensors.
Leung et al., 2015 [95]	Observational/Prospective: University Medical Centre/USA: (Elective-delirium in non-cardiac surgical patients)	n = 50, No delirium = 43/Delirium = 7	Overall—66 [11], range [43–91], No delirium—66.5 [11.1]/Delirium—70.1 [8.3]	Mini Motionlogger Actigraph (Ambulatory Monitoring, Inc., Ardsley, NY, USA	Continuous: Time1—home, 72 h; Time 2—hospital post-surgery, 72 h	Epochs of 60 s; Further processing—Action4 software—Cole–Kripke algorithm program (Ambulatory Monitoring, Inc., Ardsley, NY, USA)	Sleep: Total sleep time (night-time); Sleep onset latency (*time event marker pressed to onset of sleep*); Number of awakenings; Wake after sleep onset (%). (*Sleep diary used*) (*Bedtime and final wake times determined by the diary entry matched with a 50% change in movement during the same 10-min block of time on actigraphy*)	83% retention [60 recruited, 50 analysed]	n = 6 surgery cancelled, no hospital data, n = 4 equipment failure
^§^ Osse et al., 2009 [38]	Observational/Prospective: Medical centre/Cardiothoracic surgery dept/Holland: (Elective—Postcardiotomy delirium)	n = 79, ‘Non-cr-Del’(non-clinically relevant delirium) = 46, ‘Short-Del’ = 16, ’Sustained-Del’ = 17	‘Non-cr-Del—72.9 [4.9]/Short-Del—75.2 [4.1]/Sustained Del—75.2 [4.5]	Actiwatch (Cambridge Neurotechnology Ltd., Cambridge, UK)	Continuous: post-surgery for up to 6 days	Epochs of 60 s	Volume: Activity per minute (mean); Sedentary behaviour: Number of minutes immobile (*based on the total number of minutes with a score of zero*). Pattern:, Sedentary behaviour: number of minutes Immobile (night-time 23.00–06.00 h and daytime 06.00 h–23.00 h) Circadian Rhythm: Restlessness Index (addition of the percentage of time spent moving and the percentage immobility phases of 1 min); Activity Amplitude (difference between the least active 5 h period and the most active 10 h period within each 24-h period)	90% recruitment (eligible to recruited), 91% retention (recruited to analysed)	n = 7 technical problems
Tanev et al., 2017 [65]	Observational/Prospective: University Hospital/Psychiatry Unit/USA: (Emergency—dementia)	n = 28	82 [9]	Actigraph (Ambulatory Monitoring, Ardsley, NY, USA)	Discontinuous: 9 p.m.–7 a.m., during admission	Epochs of 60 s	Sleep: Mean sleep minutes (night-time); The deviation of each night’s sleep minutes from that mean (*Time in bed defined as 9:00 p.m. and out of bed 7:30 a.m.*)	NO	NR
Todd et al., 2017 [93]	Observational/Prospective: Teaching Hospital/Orthopedic Dept/Germany: (Elective -post-op delirium)	n = 101, Post op delirium = 27/No post op delirium = 74	Overall—76.0 [6.0], Post op delirium—75.4 [5.7]/No post op delirium—77.6 [6.7]	Actiwatch, Cambridge Neurotechnology Co., Cambridge, UK	Continuous; 1 day before op, up to 7 days after	Epochs of 60 s. Actiwatch software version 5.17 (CamNtech, Cambridge, UK)	Sleep: Total Sleep Time (night-time); Wake after sleep onset in min; Wake after sleep onset percentage (*Sleep diary used*) (*Sleep was defined as an actigraphy count of less than 80 per half-minute interval*)	77% recruitment [136 screened, 105 recruited], 85% retention [89 sensor data available]	n = 2 refused post op, n = 2 op cancelled, n = 1 died, n = 4 early discharge (n = 12 refused actigraphy, did not tolerate, or misplaced the watch during the night)
^§^ Valembois et al., 2015 [33]	Observational/Prospective/Cross sectional: Intermediate care unit/Geriatric ward/France: (Emergency—Dementia and apathy or aberrant motor behaviour)	n = 183	84.9 [6.8]	Vivago (Vivago^®^, Vivago Oy, Espoo, Finland)	Continuous: 10 days	Proprietary software (Vista, Vivago, Finland)	Pattern: Mean motor activity (grouped by pre-defined 3-h periods during the day 0:00 to 2:59, 3.00 to 05.59, 06.00 to 8.59, 9.00 to 11.59, 12.00 to 14.59; 15.00 to 17.59; 18.00 to 20.59 and 21.00 to 23.59) Sleep: Total sleep time (min); Number night awakenings (n); (Proprietary software)	100% retention (obtained analysable actigraphy data for all the patients included)	impaired sensor contact with skin/long period of time out of the detection zone*
Patients In Intensive Care									
Arttawejkul et al., 2020 [48]	Randomised Controlled Trial: Memorial hospital/Medical ICU Unit/Thailand	n = 17, Control = 9/Intervention (ear plugs, eye mask) = 8	Control median = 76, IQR = 32/Intervention median = 67, IQR = 25	Actiwatch^®^ 2 (Respironics)	Continuous: during ICU stay	Epoch of 30 s (*Further processing—by Actiware 6.0 software based on threshold of activity counts within the epoch and 2 min before and after that epoch*)	Sleep: Total sleep time (night-time); Sleep efficiency; Sleep latency; Wake after sleep onset (*Alongside Polysomnography Bedtime based on habitual bedtime at home, until 07:00*)	85% retention [20 recruited, 17 analysed]	n = 1, ICU discharge, n = 2 poor polysomnographic quality
Beecroft et al., 2008 [54]	Observational/Prospective (Validation study): General Hospital/Medical Surgical ICU/Canada: (Mechanically ventilated)	n = 12	68 [13]	Actiwatch—Model AW-64 (Philips Respironics, 1010 Murry Ridge Lane, Murrysville, PA 15668, USA)	Discontinuous: 8–12 h overnight	Sampling acceleration = 0.01 g, sampling frequency 32 Hz, epoch of 30 s: (*Further processing by Actiware-Sleep v. 3.4, software—based on threshold of activity counts*)	Sleep: Total sleep time (night-time); Sleep efficiency; Frequency of awakenings. (*Alongside Polysomnography. Frequency of awakenings—calculated at each threshold for each patient*)	NR	NR
Chen et a;., 2012 [49]	Randomised Clinical Trial: Medical Centre/Taiwan	n = 85, Experimental (valerian acupressure) = 41/Control = 44	Experimental mean—72.1 [18.2]/Control—69.1 [15.1]	ActiGraph GT1M (ActiGraph, LLC. Pensacola, FL, USA)	Discontinuous: 10 p.m.–6 a.m. (2 nights)	Processing by proprietary ActiWeb software	Sleep: Total sleep time hours (night-time); Waking minutes; Waking frequency (*Sleep based on ActiWeb software and sleep observation*)	NR	NR
Other Surgery									
Ida et al., 2019 [92]	Observational/Prospective: Tertiary Hospital/Surgical Ward/Japan: (Elective—surgical lobectomy for lung cancer)	n = 24	Overall median = 69, IQR = 4.3	WGT3X-BT (ActiGraph, LLC. Pensacola, FL, USA)	Continuous: 1 day before surgery to 6 days after	NR	Sleep: Total sleep time (night-time); Sleep efficiency (*Time tried to sleep recorded in sleep diaries, preoperative sleep efficiency < 85% was defined as acute sleep disruption*)	71% retention [24 recruited, 17 analysed]	n = 6 early discharge (within 4 days of surgery), n = 1 no sleep data for the third postoperative day
Older Adults									
^§^ Beveridge et al., 2015 [116]	Observational/Prospective: Medical Centre/Medical Ward/USA	n = 120 (Subgroup with sleep data) (Overall = 300)	Subgroup activity (with sleep data)—65.5 [11.1]	Actiwatch2, Respironics, Inc., Murrysville, PA, USA	Continuous: during admission	*Proprietary software* (Actiware 5 ‘respironics inc’—summed over 15 and 60 s intervals	Volume: Average and maximum Activity Counts (min); Total Activity Counts. (Calculated from reported wake up on morning to bed that night) Sleep: Total time spent asleep (night-time); Sleep efficiency. (*Assumed sleep period based on reported bedtimes and morning wake times*)	42% recruitment (consented to larger study to recruited), 71% retention rate [recruited to analysed]	n = 104 removed sensor at night or sensor failure, n = 49 removed during the day,
Dzierzewski et al., 2014 [115]	Observational/Prospective/Longitudinal: Veteran Affairs (VA) Medical centre/USA	n = 192, Study 1 = 85/Study 2 = 107 (intervention = 53, control = 54]	Overall—73.8 [9.4], Study 1—75 [8.5]/Study 2 intervention—73.3 [10.7]/Control—72.6 [9.5]	Octagonal Sleep Watch-L (Ambulatory Monitoring Inc.; Ardsley, New York, NY, USA)	Continuous: 7 nights/days	Epochs of 60 s. Further processing—commercially available software (ACT software, AMI)]	Sleep: Total night-time wake minutes; Daytime minutes asleep (*time in bed individually reported*)	NR for hospital data	NR for baseline data
Mixed Admission									
Alessi et al., 2008 [105]	Observational/Prospective [2 sites]: Veterans Administration Medical Centre/Community nursing home/USA: (Rehab)	n = 245, Facility A = 158/Facility B = 87	Overall—80.6 [7.2], Facility A—82.0 [7.1]/Facility B—78.1 [6.7]	Octagonal Sleep Watch-L (Ambulatory Monitoring, Inc., AMI, Ardsley, NY, USA	Continuous: 7 days	Epochs of 60 s: Further processing—commercially available software (ACT software, AMI) using time above threshold (*default algorithms*)	Sleep: Total night-time sleep (hours); Mean night-time % sleep (*hours asleep/hours between bedtime and wake-up time*); Number of night-time awakenings. Daytime hours of sleep; Daytime percent sleep. (*Night-time defined as the period between reported bedtime and reported wake-up time*)	25% recruitment, (admitted to recruited)	NR
Enomoto et al., 2010 [57]	Observational/Prospective [44 sites]: General Hospital/Mixed Acute Wards (not psychiatric or tuberculosis wards)/Japan: (Emergency)	n = 557 (analysed = 421)	Overall—72.8 [12.8], (analysed—72.5 [12.6])	Lifecorder PLUS (LC) (Suzuken, Nagoya, Japan)	Continuous: 2 days and nights	NR	Sleep: Total sleep time (night-time); Total wake time; Sleep efficiency (*Time in bed—lights out to wake approx. 9 pm–6 a.m., confirmed by nursing staff*)	76% retention (557 randomly selected to analysed)	Sudden change in physical condition/fever/severe dementia* missing data due to the amount of physical activity *
Macfarlane et al., 2019 [56]	Observational/Prospective: Hospital/Medical Assessment Ward/Australia: (Emergency)	n = 54	70.5 [17]	Sensewear actigraphy armband (Bodymedia Inc., Pittsburgh, PA, USA)	Discontinuous: 17 h—~4 p.m.–9 a.m.]	NR	Sleep: Total sleep duration (night-time mean); Sleep efficiency (*Time in bed based on lights out 10 p.m. and lights on 06:45 a.m.*)	100% retention	NR
Missildine et al., 2010 [55]	Observational/Prospective/Pilot/Multicentre [2 sites]: Acute Care/Medical Unit/USA: (Emergency)	n = 48	79	MicroMini Motionlogger (Ambulatory Monitoring, Ardsley, NY, USA)	Continuous: 24 h	Epochs of 60 s; Action-W, Version 2 software.	Sleep: Nocturnal sleep efficiency, Total sleep minutes/24 h, Total sleep minutes—night, Duration longest sleep episode—night (minutes), Number of wake episodes at night, Total wake minutes/24 h, Total wake minutes at night, Longest wake episode at night(minutes) (*Daytime sleep—scored during the hours of 7 a.m.–11 p.m.*)	Unclear	NR
Shear et al., 2014 [111]	Observational/Prospective: Academic Medical Centre/General Medicine Ward/USA	n = 424	65 [12]	Actiwatch 2 (Respironics Inc, Murrysville, PA, USA)	Continuous: up to discharge	NR	Sleep: Average sleep duration (night-time min); Sleep efficiency (%) (*rest interval, sleep onset—taken from sleep log*)	53% recruitment (eligible to recruited), 76% retention (recruited to analysed)	n = 103 removal at night or failure
Vinzio et al., 2003 [22]	Observational/Prospective: University Hospital/Acute Care Unit/France: (Emergency—cardiac, respiratory or renal acute disease)	n = 10	81 [14]	Actiwatch-L (Cambridge Neurotechnology Ltd., Cambridge, UK)	Continuous: during admission	Epochs of 60 s	Circadian Rhythm: Relative amplitude (RA)—difference between M10 and L5, M10 and L5 onset time; Interdaily stability (IS) (*perfect stability = 1, gaussian noise = 0*); Intradaily variability (IV) (*perfect sine wave = 0, gaussian noise = 2*).	80% retention (recruited to analysed)	n = 2 transferred to ICU/admitted for only 7 days, n = 2 died
Oncology									
Chang et al., 2018 [110]	Observational/Prospective: University Hospital/Taiwan: Lung cancer -newly diagnosed)	n = 84, 65 or above = 42/Below 65 = 42	Overall—65.45 [10.77], 65 or above—74.31 [6.07]/below 65–56.60 [6.12]	MicroMini Motionlogger (Ambulatory Monitoring Inc., New York, NY, USA)	Continuous: 3 days	NR	Circadian Rhythm: I < O, a percentage of activity counts less than the median activity during the rest span (*rest span is defined according to sleep–wake logbook; median of I < O* (*88.04%*) *cut-off point above this—robust rest activity rhythm, below—disrupted*)	91% retention (recruited to analysed)	n = 8 deterioration of health
^§^ Fernandes et al., 2006 [109]	Observational/Prospective/Two group comparative study: Tertiary hospital/UK: (women with cancer and matched median age group)	n = 50, patients = 25/volunteers = 25	Subject median = 67, range = [46–90]/volunteers’ median = 63, range = [54–79]	Actimeters (Ambulatory Monitoring Inc., New York, NY, USA)	Continuous: 72 h	Epochs of 60 s	Volume: Mean activity score (counts/minute) up interval—during the time awake. Sleep: % Sleep—up interval and down interval; Sleep efficiency (*% time asleep when attempting to sleep*)*;* Sleep latency (*minutes trying to sleep until first 20 min block of sleep*)*;* Wake after sleep onset (min); (*Sleep diary used to define up and down interval*) Circadian Rhythm: 24-h autocorrelation coefficient (R24) (*activity data during each 1-min ‘‘epoch’’ of a 24-h period with the activity levels during subsequent epochs*)	100% recruitment rate	NR
Jakobsen et al., 2020 [37]	Observational/Prospective: University Hospital/Palliative Medicine Dept/Norway: (Elective—advanced/metastatic cancer)	n = 40	Overall Median = 70, range (41–91)	Actiwatch 2 (Philips Respironics, Inc., Murrysville, PA, USA)	Discontinuous: 4 p.m.–9 a.m., 17 h	Epoch of 30 s. *Manufacturers’ software—medium sensitivity* (*40 activity counts*) *per epoch to score sleep, 10-min inactivity threshold for sleep onset*	Sleep: Total sleep time (night-time); Sleep onset latency; Number of awakenings during the night; Wake after sleep onset; Sleep efficiency; (*Sleep diary to establish time in bed and rest interval* (*lights off, lights on*)	32% recruitment (Screened to recruited), 98% retention (recruited to analysed)	n = 1 died, n = 2 not possible to generate sleep parameters from the Actiware software

Symbols: * No further information provided, **^§^** author/shaded row, study identified as reporting both sleep/circadian rhythm and physical activity outcomes. Abbreviations in alphabetical order: CABG = Coronary Artery Bypass Grafting, COPD—Chronic Obstructive Pulmonary Disease, IQR—interquartile range, L5—least active 5 h of the day, MET—Metabolic Equivalent of Task, M10—most active 10 h of the day, NR—Not recorded, PAEE—Physical Activity Energy Expenditure, SAVR—Surgical Aortic Valve Replacement, SD—Standard deviation, TAVI—Transcatheter Aortic Valve Implantation, THA—Total Hip Arthroplasty, TKA—Total Knee Arthroplasty, TKR—Total Knee Replacement, PFF—Proximal Femur Fracture, PHF—Proximal Humerus Fractures.
