# Supplementary material for: The Role of Wearable Sensors to Monitor Physical Activity and Sleep Patterns in Older Adult Inpatients: A Structured Review
Source: Sensors (Basel). 2023 May 18;23(10):4881. doi: 10.3390/s23104881 (PMC10222486; doi:10.3390/s23104881)
Supplement: Supplementary file 1 [file sensors-23-04881-s001.zip › sensors-2342956-supplementary.pdf]

Figure S1: The frequency of papers assessing (a) Physical activity metrics (b) Sleep/circadian rhythm metrics

(a)

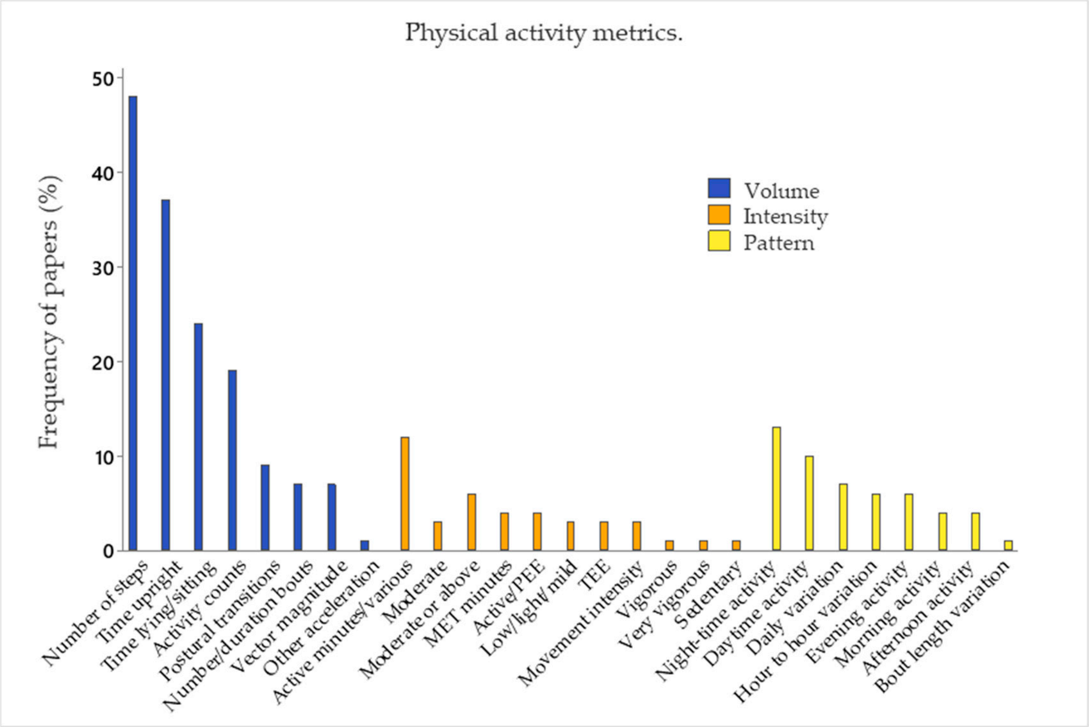

MET = Metabolic equivalent of task, PEE = Physical energy expenditure, TEE = Total energy expenditure

b)

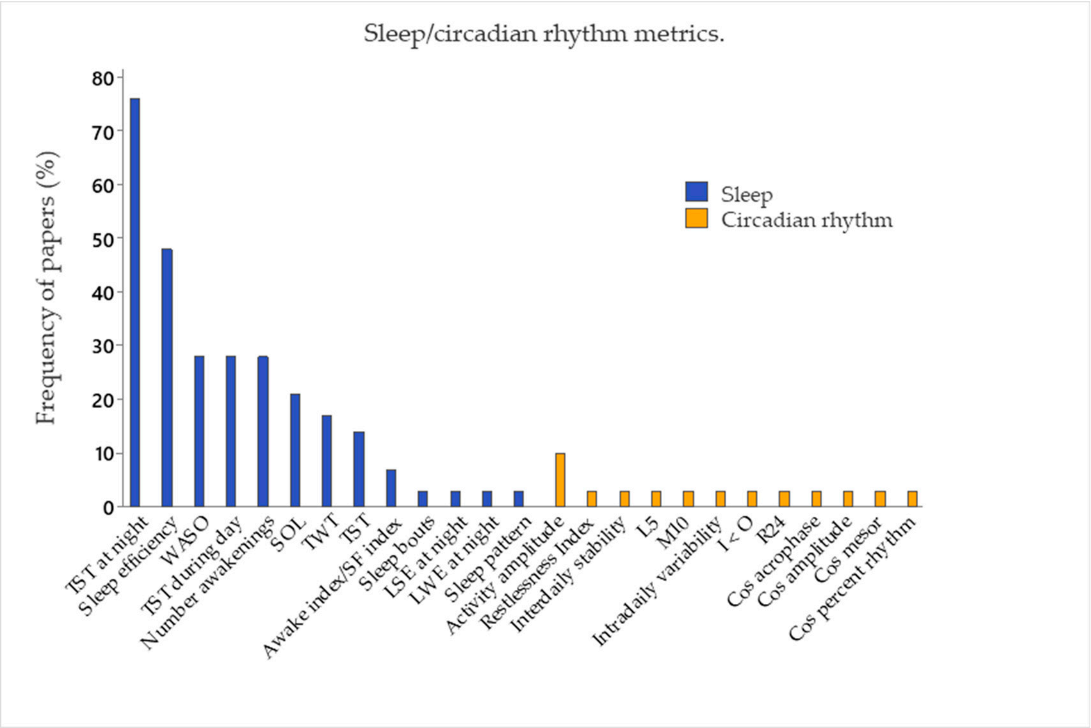

TST = Total sleep time, WASO = Wake after sleep onset, SOL = Sleep onset latency, TWT = Total wake time, TST = Total sleep time, LSE = Longest sleep episode, LWE = Longest wake episode, L5 = Lowest mean 5 hr activity, M10 = Most active 5 hr activity, R24 = 24-hour autocorrelation coefficient

Table S1: Search terms used for the review.

		Wearable sensor		Hospital		Older adult
Pubmed		**MeSH Terms:**<br><br>wearable electronic device<br>Accelerometry<br><br>**Standard Terms:**<br>smartwatch*<br>smart-watch*<br>smart watch*<br>wearabl*<br>actigraph*<br>gyroscop*<br>magnetometer*<br>inertial sensor*<br>inertial device*		**MeSH Terms:**<br><br>Hospitals<br>Critical Care<br>Surgery Department, Hospital<br>Emergency Service, Hospital<br>Hospital Units<br>Inpatients<br><br>**Standard Terms:**<br>hospita*[Title/Abstract]		**MeSH Terms:**<br><br>Aged<br>Aged, 80 and over<br>Frail Elderly<br><br>**Standard Terms:**<br><br>old*[Title/Abstract]
Ovid Embase		**TITLE-ABS-KEY:**<br>motion-sensor* or motion sensor* or motion sensor or Inertial measurement unit* or IMU or inertial-sensor* or inertial-device* or inertial device* or inertial sensor* or magnetometer* or gyroscop* or actigraph* or wearable* or smart watch* or smartwatch* or smart-watch*		**TITLE-ABS-KEY:**<br><br>"hospita*		**TITLE-ABS-KEY:**<br>Older adult* or Older or Aged or Ageing or Senior citiz* or Senior* or Frail* or Elderly or Nonagenarian* or Octogenarian* or Centenarian* or Geriatric or OAP or OAPs or Retired or Retirement or Emeritus or Gerontolog*
Scopus		**TITLE-ABS-KEY**<br>"Acceleromet*" or "Actigrap*" or "Wearable*" or "motion sensor*" or"motion-sensor*" or "Inertial measurement unit*" or "Inertial W/2 unit*" or "IMU" or "inertial-sensor*" or "inertial W/2 sensor*" or "inertial-device*" or "inertial W/2 device*" or "magnetometer*" or "gyroscop*" or "smart watch*" or "smart-watch*"		**TITLE-ABS-KEY**<br>"Hospital*" or "Psychiatric W/2 Unit*" or "Psychiatric W/2 department*" or "Medical W/2 Unit*" or "Medical W/2 department*" or "Surg* W/2 Unit*" or "surg* W/2 department*" or "critical PRE/2 care" or "critical PRE/2 department" or "rehab* PRE/2 care" or "rehab* PRE/2 department" or "intensive W/2 care*" or "icu" or "oncology*" or "hospitalised" or "hospitalized" or "outpatient*" or "inpatient*" or "out-patient*" or "in-patient*" or "secondary PRE/2 care" or "tertiary PRE/2 care "		**TITLE-ABS-KEY**<br>"old*" or "age*" or "aging" or "senior PRE/2 citiz*" or "senior W/2 adult*" or "frail*" or "elderly" or "nonagenarian*" or "octogenarian*" or "centenarian*" or "geriatric*" or "oap*" or "retir*" or "emeritus" or "gerontolog*"<br>**EXCLUDE EXACT KEYWORD**<br>"Adolescent" or "Young Adult"
Web of Science		Acceleromet* or Actigrap* or Wearable* or motion sensor* or motion-sensor* or Inertial measurement unit* or Inertial NEAR/2 unit* or IMU or inertial-sensor* or inertial NEAR sensor* or inertial-device* or inertial NEAR/2 device* or magnetometer* or gyroscop* or smart watch* or smart-watch*		Hospital* or Psychiatric NEAR/2 Unit* or Psychiatric NEAR/2 department* or Medical NEAR/2 Unit* or Medical NEAR/2 department* or Surg* NEAR/2 Unit* or surg* NEAR/2 department* or critical NEAR/2 care or critical PRE/2 department or rehab* NEAR/2 care or rehab* NEAR/2 department or intensive NEAR/2 care* or icu or oncology* or hospitalised or hospitalized or outpatient* or inpatient* or out-patient* or in-patient* or secondary NEAR/2 care or tertiary NEAR/2 care		old* or age* or ageing or aging or senior NEAR/2 citiz* or senior NEAR/2 adult* or frail* or elderly or nonagenarian* or octogenarian* or centenarian* or geriatric* or oap* or retir* or emeritus or gerontolog*
Cochran		Wearable* or motion sensor*		Hospital*		old* or age*

Table S2: Quality assessment for all included studies.

Study	Was the study population clearly specified and defined? (Age/gender/condition)	Were inclusion and exclusion criteria for participants defined? (mark no if determined after study onset)	Was the sampling method described? (e.g. random, consecutive, opportunity)	Were participants representative of the entire population they were recruited from?	Was missing data or attrition addressed with clearly defined reasons?	Was the research question or objective in this paper clearly stated?	Are the main outcomes to be measured clearly described in the Methods /Introduction. Could it be replicated?	Validated measures (criterion /convergent/discriminant validity) and implemented consistently across all study participants?	Appropriate ethics and consent?	Is the sample size justified with a power analysis and/or a study referencing a similar sample size?	Were the statistical tests used to assess the main outcomes appropriate (i.e. parametric vs. non-parametric)?	Probability values reported (e.g. 0.026 rather than <0.05) for the main outcomes. Note – unless less than 0.001	Were key potential confounding variables measured and adjusted statistically for their impact on the outcome (s)?	Aims of study fully met and clearly described?	Was reporting of results adequate (i.e. no selective reporting)	Quality Assessment 0-4 Poor 5-8 Moderate 9-12 Good ≥13 Excellent	Quality Assessment 0-4 Poor 5-8 Moderate 9-12 Good ≥13 Excellent	Quality Assessment 0-4 Poor 5-8 Moderate 9-12 Good ≥13 Excellent
**STROKE**																**Rater 1**	**Rater 2**	**Average**
Askim et al., 2013	YES	YES	YES	YES	YES	YES	NO	YES/NO	YES	NO	YES	YES	YES	YES/NO	YES	13	11	Good (12)
Bakken, 2012	YES	YES	UC	UC	YES	YES	YES	NO/YES	YES	NO	NO/YES	YES	YES	YES	YES	10	12	Good (11)
Gebruers et al., 2013	YES	YES	NO	UC	YES	YES	YES/NO	UC/NO	YES	NO	YES	NO	NO	YES	YES	9	8	Good (9)
Iacovelli et al., 2019	YES	YES	YES	UC	NO	YES	YES	UC	YES	NO	YES	YES	NO	YES	YES	10	10	Good (10)
Kerr et al., 2016	NO	YES	NO	NO	YES	YES	YES	UNCLEAR	YES	NO	YES	YES	NO	YES	YES	9	9	Good (9)
Kunkel et al., 2015	YES	NO	YES	UC	YES	YES	NO	YES	YES	NO	NO	NO/YES	NO	NO/YES	UC/YES	6	9	Moderate (8)
Norvang et al., 2018	YES	YES	UC/NO	UC	YES	YES	YES	YES	YES	NO	YES	YES	YES	YES	YES	12	12	Good (12)
Sheedy et al., 2020	YES	YES	YES	YES	YES	YES	YES	YES	YES	NO	YES	YES	NO	YES	YES	13	13	Excellent (13)

Stromm en et al., 2014	YES	YES	UC	UC	YES	YES	YES	YES	YES	YES	YES	NO	YES	YES	YES	12	12	Good (12)
**RESPIRATORY CONDITION**																		
Borges & Carvalho , 2012	Yes	Yes	Yes	Yes	Yes	Yes	Yes	Yes	Yes	Yes	Yes	No	No	Yes	Yes	13	13	Excellent (13)
Dall et al., 2019	YES	YES	YES	UC	YES	YES	NO	UC/NO	YES	YES	YES	YES	YES	YES	YES	12	12	Good (12)
Donaire-Gonzalez , 2011	Yes	No	Yes	UC	YES	YES	YES	YES	YES	NO	YES	YES/NO	YES	YES	YES	12	11	Good (12)
Orme et al., 2019	YES	YES	NO	UC	UC /NO	YES	NO/YES	UC/NO	YES	NO	UC	YES	NO/UC	YES	YES	7	8	Moderate (8)
Pitta et al., 2006	NO	NO	NO	NO	YES	YES	YES	YES	YES	NO	YES	NO	UC	YES	YES	8	8	Moderate (8)
Tsai et al., 2016	YES	YES	NO	UC	YES	YES	YES	NO/YES	YES	NO	UC	YES	NO	YES	YES	9	10	Good (10)
**CARDIAC MEDICAL/SURGICAL**																		
Amofah, 2016	YES	YES	NO	NO	NO	YES	YES	UC/YES	YES	NO	YES	YES	NO	YES	YES	9	10	Good (10)
Cook et al., 2013	YES	NO	NO	UC	NO	NO	NO	NO	YES	NO	YES	NO	NO	YES	UC	4	4	Poor (4)
Gimenez , 2017	YES	NO	YES	NO	YES	YES	NO	NO	YES	NO	YES	YES	YES	YES	YES	10	10	Good (10)
Izawa et al., 2015	YES	YES	YES	UC	NO	YES	YES	YES	YES	NO	NO	YES	NO	YES	YES	10	10	Good (10)
Mungov an et al., 2017	YES	NO/YES	YES	NO/YES	Yes	YES	YES	NO	YES	YES	UC/YES	YES/NO	YES	YES	UC/YES	10	13	Good (12)
Redeker & Wykpisz, 1999 *	YES	YES	NO	NO	NO	YES	YES	UC	YES	NO	UC	NO	NO	YES	YES	7	7	Moderate (7)
Takaesu, 2015	NO	YES	YES	NO	NO	YES	NO	NO	YES	NO	YES	YES	NO	YES	NO	7	7	Moderate (7)
Takahas hi et al., 2015	YES	NO	NO	UC	Yes	YES	UC/YES	NO	YES	NO	UC	NO	NO	YES	YES	6	7	Moderate (7)

Thorup et al., 2017	YES	YES	NO	UC	YES	YES	YES	YES	YES	NO	NO	NO	NO	YES	YES	9	9	Good (9)
Floegel et al., 2019	YES	YES	YES	UC	YES	YES	NO	YES	YES	NO	NO	NO	NO	YES	YES	9	9	Good (9)
**ORTHOPEDIC SURGERY/FRACTURES**																		
Davenport et al., 2015	YES	YES	UC/NO	UC	YES	YES	NO/YES	YES	YES	YES	UC/YES	NO	NO	NO/YES	YES	8	11	Good (10)
Denkinger et al., 2014	YES	YES	YES	UC	YES	YES	YES	YES	YES	No	YES	YES	NO	YES	YES	12	12	Good (12)
Hayashi et al., 2018	YES	YES	YES	UC	NO	YES	NO	YES	YES	YES	UC/NO	YES	NO	YES	YES	11	11	Good (10)
Keppler et al., 2020	YES	YES	YES	UC	NO	NO	YES	YES	YES	NO	NO	NO	NO	YES	YES	8	8	Moderate (8)
Krenk et al., 2013 *	YES	YES	YES	NO	YES	YES	YES	UC	YES	NO	NO	NO	NO	YES	YES	9	9	Good (9)
Kronborg et al., 2016	YES	NO	NO	UC	YES	YES	YES	YES	YES	NO	YES	YES	NO	YES	UC	9	9	Good (9)
Marsault et al., 2020	YES	YES	NO	UC	YES	YES	YES	YES	YES	YES	YES	YES	YES	YES	YES	13	13	Excellent (13)
Miller, 2015	YES	NO	NO	NO	NO	YES	NO	UC	YES	NO	YES	YES	NO	YES	YES	7	7	Moderate (7)
Peiris et al., 2013	YES	NO/UC	YES	UC	NO	YES	YES	YES	YES	NO	YES	NO	UC/NO	YES	YES	9	9	Good (9)
Schmal et al., 2018	NO	YES	NO	NO /UC	NO	YES	YES	YES	YES	YES	YES	NO	YES	YES	YES	10	10	Good (10)
Twiggs et al., 2018	YES	YES	NO	UC	YES	YES	YES	NO	YES	NO	YES	YES	YES	YES	YES	11	11	Good (11)
van Dijk-Huisman et al., 2020	YES	YES	YES	NO	YES	YES	YES	YES	YES	YES	YES	YES	YES	YES	YES	14	14	Excellent (14)
**NEUROPSYCHIATRIC**																		

Davoudi et al., 2019 *	YES	NO	NO	NO	NO	NO	YES	YES	YES	NO	YES	NO	NO	YES	YES	7	7	Moderate (7)
Evensen et al., 2019	YES	YES	NO	YES	YES	YES	YES	YES	UC	NO	YES	YES	NO	YES	YES	11	11	Good (11)
Fleiner et al., 2019	YES	YES	YES	UC/YES	YES	YES	NO/YES	YES	YES	NO	YES	YES	NO/YES	YES	YES	11	14	Excellent (13)
Jaiswal, 2020	YES	YES/NO	YES/NO	UC/YES	YES/NO	YES/NO	YES	YES /NR	YES/NR	NO/YES	YES	YES	YES	YES	YES	13	9	Good (11)
Leung, 2015	YES	YES	NO	NO/UC	YES/NO	YES	YES	UC/YES	YES	NO	YES	YES	NO	YES	YES	10	10	Good (10)
Mahlberg et al., 2007	YES	YES	YES	NO	YES	YES	YES	UC	YES	NO	UC	YES	NO	YES	YES	10	10	Good (10)
Maybrier et al., 2019	YES	UCR/NO	YES	UC/NO	YES	YES	YES	NO	YES	NO	YES	NO	YES	YES	YES	10	10	Good (10)
Osse et al., 2009 *	NO	YES	NO	UC	No	NO	YES	UC	YES	NO	YES	YES	NO	UC/NO	YES	6	6	Moderate (6)
Stubbs, 2007	NO	NO	YES	NO	UC	YES	YES	NO	NO	NO	YES	YES	NO	YES	YES	7	7	Moderate (7)
Tanev, 2017	YES	YES	YES	UC	YES	YES	YES	UC/YES	YES	NO	YES	YES	NO	YES	YES	11	12	Good (12)
Todd, 2017	YES	YES	YES	UC	YES	YES	YES	NO	YES	NO	YES	YES	YES	YES	YES	12	12	Good (12)
Valembois et al., 2015 *	YES	YES	YES	UC	NO	YES	YES	NR	UC	NO	YES	YES	NO	YES	YES	9	9	Good (9)
**PATIENTS IN ICU**																		
Arttawejkul, 2020	NO	YES	YES/NO	UC	YES	NO	NO	UC/NO	YES	YES	YES	YES	NO	NO	YES	8	7	Moderate (8)
Beecroft, 2008	YES	YES	YES	UC/NO	NO	YES	YES	UC/NO	YES	NO	UC/YES	YES	NO	YES	YES	9	10	Good (10)
Chen, 2012	NO	YES	YES	UC/NO	NO	YES	YES	UC	YES	YES	NO	YES	NO	YES	YES	9	9	Good (9)
Estrup et al., 2019	YES	NO	YES	UC	YES	YES	YES	UC	YES	NO	YES	YES	NO	YES	UC	9	9	Good (9)

**OTHER SURGERY**																		
Matsuo et al., 2015	YES	NO	NO	UC	UC	YES	NO	UC	YES	NO	YES	NO	NO	YES	YES	6	6	Moderate (6)
Ida, 2019	YES	YES	NO	UC	YES	YES	YES	UC	YES	NO	YES	NO	NO	YES	NO	8	8	Moderate (8)
Jonsson et al., 2019	YES	UC/NO	YES	UC	YES	YES	YES	YES	YES	NO	YES	YES	YES	YES	YES	12	12	Good (12)
**OLDER ADULTS**																		
Beveridge et al, 2015 *	YES	YES	YES	UC	YES	YES	YES	NO	YES	NO	YES	YES	YES	YES	YES	12	12	Good (12)
Brown et al., 2009	YES	YES	YES	NO	NO	YES	NO	YES	YES	NO	NR	NR	NO	YES	YES	8	8	Moderate (8)
Cohen et al., 2019	YES	YES	YES	NO -	YES	YES	NO	NO	YES	NO	YES	YES	YES	YES	YES	11	11	Good (11)
Dzierzewski, 2014	YES	YES	NO	UC	NO	YES	YES	YES	YES	NO	YES	YES	YES	YES	YES	11	11	Good (11)
Evensen et al., 2017	YES	YES	YES	UC	NO	YES	YES	YES	YES	NO	YES	YES	YES	YES	YES	12	12	Good (12)
Fisher, 2011	YES	YES	YES	NO	YES	YES	YES	YES	YES	NO	YES	YES	NO	YES	YES	12	12	Good (12)
Klenk et al., 2019	YES	NO	NO	UC	NO	YES	YES	YES	YES	NO	NO	NO	NO	YES	YES	7	7	Moderate (7)
Kolk et al., 2021	YES	YES	NO	YES	YES	YES	YES	UC	YES	NO	YES	YES	YES	YES	YES	12	12	Good (12)
Lim et al., 2018	YES	YES	NO	UC	No	YES	YES	YES	YES	NO	YES	YES	YES	YES	YES	11	11	Good (11)
Hartley, 2018	NO/YES	YES	YES	NO	YES	YES	YES	YES	YES	YES	YES	NO	NO	YES	YES	11	12	Good (12)
McCullagh, 2016	NO/YES	YES	YES	NO	YES/NO	YES	YES	YES	YES	NO	YES	NO	YES	YES	YES	11	11	Good (11)
Moreno et al., 2019	YES	YES	YES	YES	YES	YES	YES	UC	YES	YES	UC	NO	NO	YES	YES	11	11	Good (11)

Norheim et al., 2017	YES	YES	UC	NO	YES	YES	NO	UC/NO	YES	NO	YES	NO	NO	YES	YES	8	8	Moderate (8)
Ostir et al., 2013	YES	YES	NO	UC	YES	YES	YES	YES	YES	NO	YES	NO	YES	YES	YES	11	11	Good (11)
Pedersen et al., 2013	YES	YES	YES	NO/UC	YES	YES	YES	YES	YES	NO	YES	YES	YES	YES	YES	13	13	Excellent (13)
Tasheva et al., 2020	YES	YES	YES	UC	NO	YES	YES	YES	YES	NO	YES	YES	YES	YES	YES	12	12	Good (12)
Theou et al., 2019	YES	UC	YES	YES	UC	YES	YES	YES	YES	NO	YES	YES	YES	YES	YES	12	12	Good (12)
**MEDICAL/MIXED ADMISSION**																		
Alessi, 2008	YES	YES	YES	UC	NO	YES	YES	YES	YES	NO	YES	YES	YES	YES	YES	12	12	Good (12)
Chaboyer et al., 2015	NO	YES	YES	NO -	NO	YES	YES	YES	YES	NO	NO	NO	NO	YES	NO/YES	7	8	Moderate (8)
Enomoto, 2010	NO	NO	YES	YES	NO	YES	YES	UC	YES	NO	UC	NO	YES	YES	YES	8	8	Moderate (8)
Fisher et al., 2016	YES	YES	NO	UC	YES/NO	YES	YES	YES	YES	NO	YES	NO	YES	YES	YES	11	11	Good (11)
Macfarlane, 2019	YES	YES	NO	NO	NO	YES	NO	UC	YES	NO	NO	NO	NO	YES	YES	6	6	Moderate (6)
Missildine, 2010	NO	YES	YES	NO	NO	YES	YES	YES	YES	NO	YES	YES	UC	YES	Yes	10	10	Good (10)
Sallis et al., 2015	YES	NO	NO	YES	NO	YES	NO	YES	YES	NO	YES	YES	UC/NO	YES	YES	9	9	Good (9)
Shear, 2014	YES	YES	YES	NO	YES	YES	YES	UC	YES	NO	YES	YES	YES	YES	YES	12	12	Good (12)
Vinzio, 2003	NO	NO	NO	NO	YES	NO	YES	NO	YES	NO	YES	YES	NO	YES	UC	6	6	Moderate (6)
**PATIENTS WITH CANCER**																		
Chang, 2018	YES	YES	YES	UC	YES	YES	YES	UC	YES	NO	YES	YES	YES	YES	YES	11	11	Good (11)

Jakobsen, 2019	YES	NO	NO	NO/UC	YES	YES	YES	UC	YES	YES	YES	NO	NO/YES	YES	YES	9	8	Good (9)
Jonker et al., 2020	YES	YES	NO	NO	Yes	YES	YES	UC/NO	YES	NO	YES	YES	NO	YES	YES	10	10	Good (10)
Porserud et al., 2019	YES	YES	NO	YES	NO	YES	YES	YES	YES	YES	YES	YES	NO	YES	YES	12	12	Good (12)
Morikawa et al., 2018	YES	YES	NO	UC	YES	YES	NO/YES	NO	YES	NO	UC/NO	NO	NO	YES	NO/UC	6	7	Moderate (7)
Fernandes et al., 2006 *	YES	NO	YES	NO	NO	YES	YES	YES	YES	NO	YES	YES	YES	YES	UC	10	10	Good (10)
**PARKINSONS DISEASE**																		
Ito et al., 2020	YES	YES	NO	UC	NO	NO	YES	YES	YES	NO	YES	YES	NO	YES	YES	9	9	Good (9)

Abbreviations: UC = Unclear. Rating: YES = scored as 1, NO or UC = scored as 0.

Author * identifies studies including both physical activity and sleep/circadian rhythm outcomes.

Table S3: The sensor models and versions used to assess physical activity and sleep/circadian rhythm outcomes

Sensor Model	Number of physical activity studies (n)	Number of sleep/ circadian rhythm studies (n)
**ActivPAL** - Versions: ActivPAL/ ActivPAL2/ActivPAL3/ ActivPAL3 micro	**15**	**0**
**ActiGraph** - Versions: wActiSleep Plus/ GT1M (uniaxial) / GT3X/WGT3X-BT	**6**	**3**
**Fitbit** - Versions: The Zip / Charge2 / Flex	**5**	**0**
**Motionlogger** (Ambulatory Monitoring Inc) – Versions: Octagonal basic motion loggers/ Octagonal sleep watch - L /The Mini Motion Logger/Micro SleepWatch	**4**	**9**
**Actiwatch** (Philips Respironics) - Versions: Actiwatch2/Actiwatch spectrum plus/ AW-64/Actical	**4**	**9**
**SenseWear Armband** - Versions: Pro2/Pro 3/Bodymedia	**4**	**1**
**StepWatch Activity Monitor (SAM)**	**4**	**0**
**Lifecorder** - Versions: EX 1-axial accelerometer/ GS / Plus	**3**	**1**
**Axivity™** - Version: AX3	**3**	**0**
**Active Style Pro** – Version: HJA-350IT	**3**	**0**
**Actiwatch** (Cambridge Neurotechnology Ltd) - Version: Actiwatch-L	**2**	**3**
**DynaPort Moviemonitor**	**2**	**0**
**AugmenTech, Inc**	**2**	**0**
**Tractivity®**	**2**	**0**
**GENEActiv**	**2**	**0**
**Vivago**	**1**	**1**
**EZ430-Chronos**	**1**	**0**
**Shimmer** – Version: Shimmer 3 (GYR)	**1**	**0**
**Physilog ® (GYR)**	**1**	**0**
**Actibelt ®**	**1**	**0**
**Misfit Shine**	**1**	**0**
**MOX**	**1**	**0**

**uSense**	**1**	**0**
**StepWatch**	**1**	**0**
**ADXL362** (Analog devices inc)	**1**	**0**
**No details**	**2**	**2**

Abbreviations: GYR = Gyroscope
